# Patient-derived organoids for precision oncology: a platform to facilitate clinical decision making

**DOI:** 10.1186/s12885-023-11078-9

**Published:** 2023-07-22

**Authors:** Swati Chitrangi, Pooja Vaity, Aishwarya Jamdar, Shweta Bhatt

**Affiliations:** 1 Department of Integrated Drug Discovery and Development, Yashraj Biotechnology Limited, C-232 and C-113, TTC Industrial Area, MIDC, Pawane, Maharashtra 400705 India; 2 Yashraj Biotechnology GmbH, Uhlandstraße 20-25, 10623 Berlin, Germany; 3 Yashraj Biotechnology Limited, 8, The Green STE A, Dover, Delaware State 19901 USA

**Keywords:** Patient-Derived Organoids (PDOs), Tumor Microenvironment (TME), Patient-derived Xenograft (PDX), Drug efficacy and safety studies, Patient-derived induced pluripotent stem cells (iPSCs)

## Abstract

**Background:**

Despite recent advances in research, there are still critical lacunae in our basic understanding of the cause, pathogenesis, and natural history of many cancers, especially heterogeneity in patient response to drugs and mediators in the transition from malignant to invasive phenotypes. The explication of the pathogenesis of cancer has been constrained by limited access to patient samples, tumor heterogeneity and lack of reliable biological models. Amelioration in cancer treatment depends on further understanding of the etiologic, genetic, biological, and clinical heterogeneity of tumor microenvironment. Patient-derived organoids recapitulate the basic features of primary tumors, including histological complexity and genetic heterogeneity, which is instrumental in predicting patient response to drugs.

**Methods:**

Human iPSCs from healthy donors, breast and ovarian cancer patients were successfully differentiated towards isogenic hepatic, cardiac, neural and endothelial lineages. Multicellular organoids were established using Primary cells isolated from tumor tissues, histologically normal tissues adjacent to the tumors (NATs) and adipose tissues (source of Mesenchymal Stem Cells) from ovarian and breast cancer patients. Further these organoids were propagated and used for drug resistance/sensitivity studies.

**Results:**

Ovarian and breast cancer patients’ organoids showed heterogeneity in drug resistance and sensitivity. iPSCs-derived cardiomyocytes, hepatocytes and neurons showed donor–to-donor variability of chemotherapeutic drug sensitivity in ovarian cancer patients, breast cancer patients and healthy donors.

**Conclusion:**

We report development of a novel integrated platform to facilitate clinical decision-making using the patient's primary cells, iPSCs and derivatives, to clinically relevant models for oncology research.

**Supplementary Information:**

The online version contains supplementary material available at 10.1186/s12885-023-11078-9.

## Background

The incidence of cancer is increasing globally, posing a significant challenge in developing effective and economically viable anticancer drugs. Clinical approval for drugs entering phase I clinical trials after preclinical studies is crucial, yet the current rate of clinical approval for potential anticancer drugs is as low as 5%, much lower than drugs for other diseases [1]. Identifying a safe, potent and efficacious drug requires thorough preclinical testing, which evaluates aspects of pharmacodynamics, pharmacokinetics, and toxicology in in vitro and in vivo settings [2]. This necessitates the development of more effective preclinical platforms for screening anticancer compounds. In vitro and patient-derived tumor models are essential for identifying substances with anticancer activity and evaluating their effectiveness. Physiologically relevant in vitro models enable detailed primary screening, preventing inadequate drugs from progressing to animal testing [3].

While animal models are currently used in preclinical studies, they present challenges such as high cost, species-specific responses, and limitations in availability and feasibility (e.g. ethical issues, extended timelines of development, poor human disease recapitulation). Mammalian models, such as pig, rabbits, etc., have been regarded as the gold standard for assessing drug toxicity due to similarity in metabolic enzymes and pathways. Species differences in drug penetration of the blood–brain barrier, drug metabolism, and related toxicity contribute to failure of drug trials from animal models to human [4]. Therefore, there is a need to create advanced in vitro models to assess bioavailability and therapeutic efficacy of anticancer drugs [5].

Tumor is a highly heterogeneous disease; different types of cancers usually differ in clinical features and tumorigenic mechanisms, leading to different responses to drugs [6]. How to accurately predict a patient's response to drugs and choose a personalized treatment is one of the main challenges for clinicians. Adding to the complexity, cancer cells are known to be embedded in a tumor microenvironment (TME) composed of stromal cells (MSCs or fibroblast), endothelial cells and extracellular matrix (ECM) [7]. The TME is critical for promoting tumor growth, has a direct role in tumor progression and metastasis and contributes significantly to the resistance to therapy [8]. Mechanisms of drug or therapy resistance include involvement of cancer-associated fibroblasts (CAFs) in driving synthesis and cross-linking of large amounts of collagens to form a fibrotic ECM, reducing tumor access of cytotoxic drugs and activation of integrin-mediated mechano-transduction in cancer cells [9]. Drug testing modalities with conventional monoculture preclinical models is misleading and leads to high failure rate in more advanced stages like phase 3 clinical trials [10]. It is therefore imperative to develop reliable model(s) that are suitable in predicting patient response and enable personalized treatment strategies. Although the development of disease staging, sub-typing, sequencing, molecular and immuno phenotyping helps in clinical management, effective tools are needed to predict individual’s response to any given therapy, owing to large variations driven by genetic and environmental heterogeneity. Culturing cancer organoids could help in patient specific response profiling. In addition, organoids cultured from patient tissues have been shown to preserve the tissue architecture, gene expression profile of the original cancers. Patient-derived tumor organoids better recapitulate native tumors and may be good models for identifying and testing new anticancer drugs as well as response to existing treatment regimes in a personalized setting [11].

By combining tumor organoids and healthy organoids from one patient to assess therapeutic effects, we can choose the best therapy that selectively kills tumor cells and leaves healthy cells undamaged [11]. Second, patient-derived organoids (PDOs) are an ideal platform for high-throughput anticancer drug screening as well as for modeling metastasis, tumor heterogeneity, and to some extent the TME. They have great potential for disease modeling for cancer research, and living biobanks of various types of cancer organoids are warranted for capitalizing on the promise of this technology.

Treatment of cancer has evolved in the last decade with recent advances in the processing and culture of human tissue, bioengineering, xenotransplantation, genome editing and induced pluripotent stem cells (iPSCs) technology. Despite these successes, the lingering drug toxicity side-effects from chemotherapy remain a major cause of morbidity and mortality in cancer survivors. Chemotherapeutic agents aim to damage rapidly proliferating cancerous cells. However, most chemotherapeutic agents cannot distinguish between malignant and healthy cells, leading to toxicities in healthy organs. These toxicities can manifest acutely or chronically, even after chemotherapy is completed, and can affect various organs such as the heart [12], liver [13], kidneys [14], gastrointestinal system [15], lungs [16], bone marrow [17], and nervous system [18], leading to dangerous complications and negative patient outcomes if not adequately understood and assessed.

Alkylating agents like Cyclophosphamide, Melphalan, Chlorambucil, Procarbazine induce apoptosis in liver cells mediated by tumor necrosis factor (TNF) released by Kupffer cells [19]. Nitrosoureas such as carmustine and lomustine as well as Streptozotocin, produce alkylation and disruption of DNA and RNA, cause hepatic necrosis. An antibody–drug conjugate, Trastuzumab results in serious hepatotoxicity, including liver failure and death [20]. Methotrexate often elevates transaminase levels in 60% to 80% of patients; when used for long-term, it can also cause fibrosis and cirrhosis [21]. Doxorubicin is known to cause liver toxicity as a result of idiosyncratic reactions [22].

Several chemotherapeutic drugs induce side-effects such as growth impairment and suppression of angiogenesis resulting in severe apoptosis and necrosis, leading to damage of the myocardium and severe cardiotoxicity. Anthracyclines, is one such class of chemotherapeutic agents, that cause severe cardiotoxicity manifested largely through mitochondrial damage, apoptosis and changes in ATP and free radical production [23]. Trastuzumab potentiates the cardiotoxic effect of anthracyclines, via interference with ErbB2 receptor signaling pathways [24]. Alternatively, induced cardiotoxicity could be associated with myocardial damage via effects on subcellular organelles, increased histamine release, resulting in arrhythmias, as seen induced by Taxanes [25]. 5-Fluorouracil has direct toxic effects on vascular endothelium, leading to coronary spasm and endothelial-independent vasoconstriction via protein-kinase C [26]. Doxorubicin (adriamycin), daunorubicin, and epirubicin are anthracyclines known to weaken myocardial contractility [27].

Vincristine, vinblastine, procarbazine and cisplatin cause neuropathies with accompanying paresthesia, loss of deep tendon reflexes, and muscle weakness [28]. Cisplatin, along with its renal toxicity, may affect the nervous system. Methotrexate, cytarabine (cytosine arabinoside) and ifosfamide are also primarily known for their neurotoxic side effects [29].

The development of induced pluripotent stem cell (iPSC) technology in 2007 has enabled the development of a new human technology platforms (e.g. organoids or organ-on-a-chip) for disease modeling and drug screening through in vitro/ex vivo testing of drugs for efficacy and safety. iPSC technology has opened a new avenue for scientists which allows unlimited access to human hepatocytes [30], cardiomyocytes [31], endothelial cells [32], neurons [33] and other cell types involved in drug toxicity. Research on a variety of diseases, including rare diseases and those with multifactorial origins, as well as to simulate drug effects on difficult-to-obtain tissues like the brain and cardiac muscles, has been made possible by the growing number of human disease models created with patient-specific iPSCs. Before advancing to clinical trials, toxicity and teratogenicity studies performed with iPSC-derived cells can add an extra level of assurance. The active role of human iPSC-derived in vitro models has been identified in phenotypic screening, target-based screening, target validation, toxicology evaluation, precision medicine, "clinical trial in a dish," and post-clinical studies by examining each stage of the drug discovery and development processes. Advantages of iPSCs include their patient origin, easy availability, expandability, ability to give rise to almost any desired cell type, avoidance of ethical concerns associated with human embryonic stem cells (hESCs), and potential to develop personalized medicine [34]. Human iPSCs-based models are low-cost, simple, convenient, and imitate in vivo organ microenvironment. Mature cell lineages differentiated from human iPSCs can construct organoid models that have homology with the donor without species differences. We are no longer restricted to a single type of cell since we can fully replicate the process of drug metabolism in the organ to assess the impact of medications on a range of cells developed by iPSC technology. Organoid models can be developed in a petri dish, require less starting material, and cost less and have thus become a model of choice in many drug development pipelines [35]. 

iPSCs can be used to establish associations between genotype and drug responses and to identify biomarkers which can also assist in patient selection and/or stratification in clinical trials. For example, pharmacological reversal of the hyperexcitability phenotype in iPSC-derived sensory neurons from patients with pain disorder, hereditary erythromelalgia, by a selective sodium channel blocker has been found to correlate with patient responses and patient-specific mutations [36]. Finally, patient-derived iPSCs can also be envisioned as accompanying diagnostics or as avatars to guide treatment tailored to individual patients. A major challenge in these applications is to match the time scales of model building with the time scales of clinical decision making. The establishment of iPSCs could be advantageous in this regard, as the time from somatic cell harvest to derivation of the appropriate cell model is on average 2–3 months compared to longer than 6 months for PDX models [37].

While the field of iPSC-derived cancer research is still evolving from its infancy, we have developed patient-derived iPSC lines to model cancer-predisposing disorders. Our current study highlights the potential of human iPSCs in cancer studies by overcoming limitations related to the availability of patient samples or the translation of results from animal models or cell lines with inappropriate genetic backgrounds. These iPSC models will also be used to investigate the mechanisms of oncogenesis and cancer progression. We have also established robust protocols to directly differentiate these human iPSCs into hepatocytes (liver cells), cardiomyocytes (heart cells), neural progenitor cells (brain cells), astrocytes, midbrain dopaminergic neurons, forebrain motor neurons and endothelial cells in 2D as well as 3D culture. Furthermore, we have also established patient derived organoids (PDOs) from tumor and adjacent normal tissues co-cultured with the patient's Mesenchymal Stem Cells (MSCs) and iPSCs derived endothelial cells to recapitulate the in vivo tumor microenvironment. These PDOs recapitulate epithelial architecture and model tumor heterogeneity, making them a promising tool for studying drug resistance and efficacy or for identifying new drug discovery in a relatively short period of time. In addition, these patient-derived iPSCs and derivatives are suitable for high-throughput screening (HTS), cryopreservation and long-term maintenance. Together with methods for reprogramming patient-derived iPSCs, differentiating these iPSCs into disease-relevant cell types, organoids derived from patient tissues, we have developed a fully integrated platform for in vitro*/ *ex vivo disease modeling for accelerated clinical testing and discovery. These models can be used for target identification, lead optimization and validation, drug repurposing, small molecule screening, toxicity studies as well as dose ranging etc. during the drug development process.

## Methods

### Ethical statements for studies involving human samples and patient informed consent

The study was approved by the Institutional Committee for Stem Cell Research (IC-SCR) duly registered with National Apex Committee for Stem Cell Research and Therapy (NAC-SCRT) of ICMR registration ID: NAC-SCRT/134/20200209 and The Institutional Ethics Committee (IEC) duly registered with Drug Controller General of India (DCGI) registration ID: ECR/305/Indt/MH/2018. Signed voluntary informed consent was obtained from the patients. The animal experiments were conducted after approval from Institutional Animal Ethics Committee (IAEC) of iSERA Biological Pvt Ltd, India registered with Committee for the Purpose of Control and Supervision of Experiments on Animals (CPSEA) via registration no. ISERA/IAEC/S/2022/08. Biospecimens are traceable and are uniquely identifiable by a coding system that protects the donors’ identity, thereby blindfolding the experimenters who perform research (to avoid bias). The informed patient consent document allows the use of the biological material for research, development and manufacturing.

### Sample processing and establishment of patient-derived cells

A total of 41 ovarian cancer and 80 breast cancer patients’ biospecimens (i.e. blood-with anticoagulant, blood-without anticoagulant, urine, tumor, normal adjacent tissues and adipose tissues) were collected from patients (Inclusion criteria: Age ≥ 18 years) at Om Sai Onco Surgery Hospital, Maharashtra, India and Sushrut Hospital (and allied multi-specialty hospitals), Maharashtra, India between January 2019 to April 2022. All samples were transported from the hospital to research and development laboratories of Yashraj Biotechnology Ltd. at Maharashtra, India via validated cold chain logistics for downstream processing and R&D (sample details in Supplementary Data Table 2).

- **Serum:** Blood without anticoagulant was allowed to clot for 30 min and clot was removed by centrifuge at 1,000–2,000 × g for 10 min in a refrigerated centrifuge. Extracted serum was aliquoted and stored. Serum is suitable for biomarker (genomics, proteomics) studies.
- **Urine:** Urine sample was centrifuged in a refrigerated centrifuge (4^0^ C) at 1500 g for 10 min to remove sediments. Urine supernatant and pellet were aliquoted and stored. Urine is a major repository of biometabolites, some proteins, and DNA. Urine is suitable for biomarker (metabolomics) studies.
- **Peripheral Blood Mononuclear Cells (PBMCs):** Blood with anti-coagulant was diluted 1:1 (vol:vol) in DPBS and layered over Histopaque (sigma). PBMCs were isolated by Histopaque density gradient centrifugation method and cryopreserved (post viability determination by Trypan Blue dye exclusion method). PBMCs are suitable for reprogramming for iPSC development, T-cell engineering, immune cells research, cell-line development, immunophenotyping assays etc.
- **Tissue snap-freezing:** Tumor and normal adjacent tissues (from isogenic donor) were cut into small pieces and tissues no thicker than 0.4 cm longitudinal section were aliquoted in vials and snap frozen in liquid nitrogen (for 2 min or less depending on the size of the tissue) and stored in -80^0^ C freezer for long-term storage. Snap-frozen tissues can be used for downstream analysis of DNA, RNA and protein. 2 mm^3^ tissue tissues were also cryopreserved in 10% DMSO and 90% FBS which is suitable for developing patient derived xenograft (PDX) models for in vivo pharmacology studies.
- **Formalin-Fixed Paraffin Embedded (FFPE) Tissue Block preparation:** Tumor and normal adjacent tissues were cut into small pieces and tissues no thicker than 0.4 cm longitudinal section were fixed in 10% Neutral Buffered Formalin (NBF), followed by serial dehydration in ethanol, tissue clearing in xylene and embedding in paraffin. Formalin-Fixed Paraffin Embedded (FFPE) blocks were stored long-term for histopathology, biomarker testing.
- **Establishing a Collection of Patient-Derived Breast Cancer and Ovarian Cancer Organoids:** At the time of each patient’s surgical debulking (i.e. “surgical waste”), Tumor and histologically Normal-appearing Adjacent-to-Tumor (NAT) tissues were collected and mechanically dissected followed by enzymatic treatment. Minced tissues were incubated in enzymatic degradation solution containing Advanced DMEM: F12 (Gibco), collagenase I (Sigma), dispase II (Sigma), Rock Inhibitor- Y-27632 (Sigma), and DNase I (Stem Cell Technologies). The mixture was incubated in shaking waterbath at 37^0^C, shaken at 180–200 rpm for 30—90 min. After incubation mixture was passed through 70 µm cell strainer (Corning). Cell suspension was centrifuged at 300 g for 10 min. Cells were counted and seeded in 96 well plate(s) at cell density of 1 × 10^4^ cells per well in matrigel. Cells were overlaid with an optimized culture medium containing critical compounds and growth factors that allow the generation of breast and ovarian organoids. Breast organoid medium consists of Advanced DMEM:F12 (Gibco) supplemented with 1X Glutamax, 1X B27 supplement, 5% FBS, 100 ng/mL Noggin, 20 ng/mL EGF, 50 ng/ml cholera toxin, 0.5 µg/ml hydrocortisone and 10 µg/ml insulin. Ovarian organoid medium consists of MCDB 105 Medium (Sigma), Medium 199 Earle's Salts (Thermo Fisher Scientific), 1 × GlutaMAX-I (Thermo Fisher Scientific), 1X B27 supplement minus vitamin A (Thermo Fisher Scientific), 100 ng/mL Noggin (PeproTech), 10% FBS, 50 ng/mL EGF and 10 µg/ml insulin. Moreover, 10 µM Y-27632 was added to culture media for the first three days of culture. Medium was changed every 4 days and organoids were passaged every 1–4 weeks. These patient-derived organoids are suitable for drug efficacy and safety studies.
- **Mesenchymal Stem Cells (MSCs) isolation from Adipose Tissue:** The Adipose tissue was cut with scalpels into pieces of about 1 mm thickness in fresh dissection medium, plated in DMEM/F-12, supplemented with 10% FBS. Once MSCs outgrowth formed around most explants, the explants were removed and the culture was continued in DMEM/F-12 (Gibco) supplemented with 10% FBS (Gibco). Primary cultures were passaged and characterized by immunophenotyping using Human MSC Analysis Kit (BD Biosciences) for CD73, CD105 and CD90 cell surface markers. These MSCs are suitable for development of patient-derived organoids.

### Generation of human iPSCs by Sendai viral reprogramming of PBMCs

PBMCs were cultured in StemPro-34 serum-free medium (Stem Cell Technologies) supplemented with 100 ng/mL SCF (Stem Cell Technologies), 100 ng/mL FLT-3 (Stem Cell Technologies), 20 ng/mL IL-3 (Stem Cell Technologies), 20 ng/mL IL-6 (Stem Cell Technologies) prior to reprogramming with Cytotune™-iPS 2.0 Sendai virus (ThermoFisher Scientific) expressing OCT4, SOX2, KLF4 and cMYC (Bhatt et al. [38] and Chitrangi and Bhatt et al., [39–41]), in the presence of 4 µg/ml Polybrene (EMD Millipore). Transduced cells were plated on vitronectin (Stem Cell Technologies) coated plates. iPSC colonies were mechanically cut and further expanded in mTeSR™ (Stem Cell Technologies). Human iPSCs were characterized by expression of Oct4, Nanog, Sox2, SSEA4 using flowcytometry and immunocytochemistry. These human iPSCs are suitable for directed differentiation, disease modeling, CRISPR-Cas9 mediated gene editing, developmental biology, drug discovery applications, organoid development and precision drug screening etc.

### Karyotype and Short tandem repeat (STR) analysis

Karyotypes and STR analysis was performed by Medgenome Labs Private Limited [39–41] to evaluate genomic stability and purity.

### Teratoma assay

Teratoma assay was performed as per [38]. Briefly, approximately 2 × 10^6^ cells harvested as cell clumps, in a 5:1 mixture with Matrigel (BD Biosciences) were injected intramuscularly in NOD SCID male mice 12–18 weeks old. Animals were monitored for cellular engraftment and in vivo growth and differentiation over the next 4–8 weeks while the teratomas started to emerge/grow, and the size measurements were recorded every week using Vernier calipers. Animals were procured from Advanced Centre for Treatment, Research and Education in Cancer (ACTREC), Maharashtra, India and sacrificed using CO_2_ asphyxiation.

### In vitro hepatic differentiation of human iPSCs

To initiate hepatic differentiation, 10, 000 cells/cm^2^ of passage 11 (P11) single cell suspension of iPSCs were seeded on matrigel coated plates in mTeSR™1 media and incubated overnight to obtain 95% confluent culture. Once cells reached > 95% confluence, RPMI media (Gibco) + 2% B27 supplement (Gibco) + 100 ng/ml Activin A (Gibco) + 3 µM CHIR99021 (Stem Cell Technologies) + 2% Matrigel (Corning) was added. Day 1 to 4: RPMI (Gibco) media + 2% B27 supplement (Gibco) + 100 ng/ml Activin A (Sigma). Day 5 to 10: RPMI (Gibco) media + 2% B27 supplement (Gibco) + 10 ng/ml bFGF (Sigma) + 20 ng/ml BMP4 (Sigma) + 0.5% DMSO (Sigma); Day 11 to 15: RPMI 1648 (Gibco) media + 2% B27 supplement (Gibco) + 20 ng/ml HGF (Sigma) + 0.5% DMSO (Sigma); Day 16 to 21: RPMI 1648 (Gibco) media + 2% B27 supplement (Gibco) + 20 ng/ml Oncostatin M (Gibco) + 100 nM Dexamethasone + 20 ng/ml HGF (Sigma) + 0.5% DMSO (Sigma). These hepatocytes were characterized by expression of albumin using flowcytometry and immunocytochemistry and were further tested for gene expression analysis, subjected to drug screening, disease modeling, metabolic analyses, hepatotoxicity assays, viral infectivity studies, glucose regulation studies, transporter function studies and various other downstream assays. The presence of intracellular glycogen in hepatocytes was assessed by periodic acid–Schiff’s (PAS) staining and nuclei were counterstained with Mayer’s haematoxylin [42].

### In vitro cardiac differentiation of human iPSCs

To initiate cardiac differentiation, 10,000 cells/cm^2^ of passage 11–20 single cell suspension of iPSCs were seeded on matrigel coated plates in mTeSR™1 media and incubated overnight to obtain 95% confluent culture. Once cells reached > 95% confluence, RPMI 1648 medium (Gibco)/B-27 Supplement Minus Insulin (Gibco)/ 100 ng/ml Activin A (Gibco) + Matrigel (Corning) [43] + 3 µm CHIR9902 (Stem Cell Technologies) was added. CHIR9902 only for 24 h. Day 02–04: RPMI 1648 medium (Gibco)/B-27 Supplement Minus Insulin (Gibco)/ activin A (Gibco) (100 ng/ml) and BMP4 (Gibco) (10 ng/ml) and bFGF. Day 05–06: RPMI 1640 (with L-glutamine) with 2% B27 minus insulin supplement, 1 µl IWR-1 (5 µM final). Day 07–09: RPMI 1640 with L-Glutamine (Gibco) with 2% B27 with insulin supplement (Gibco). Day10: At day 10 post-differentiation, changed the medium to RPMI 1648-low glucose medium (Gibco) and maintained the cells in this medium for 3 days (until day 13). Day 13: Returned cells to RPMI/B27 medium-with insulin (Gibco). At day 14, changed the medium back to RPMI-low glucose medium (Gibco) for a second glucose deprivation cycle. Cultured the cells in this low glucose state for 3 more days. Most of the non-cardiomyocytes will die in this low-glucose culture condition. At day 17, changed the medium to 2 ml of RPMI/B27 medium with insulin. The remaining cells will be highly purified cardiomyocytes. These cardiomyocytes were characterized for the expression of cardiac troponin (cTnT) using flowcytometry and immunocytochemistry and further tested for gene expression analysis, subjected to drug screening, metabolic analysis, cardiotoxicity assays, disease modeling, transporter function studies and various other downstream assays. Cardiotoxicity was analyzed by calcium transient assay using ImageXpress® Nano Automated Imaging System, Molecular Devices [44, 45].

### In vitro endothelial cell differentiation of human iPSCs

To initiate endothelial cell differentiation, 10,000 cells/cm^2^ of passage 11–20 single cell suspension of iPSCs were seeded on matrigel (Corning) coated plates in mTeSR™1 media and incubated overnight to obtain 95% confluent culture. Once cells reached > 95% confluence, N2B27 medium supplemented with 8 µM CHIR-99021 and 25 ng/mL hBMP4 was added. Day 4–6, StemPro-34 medium supplemented with 300 ng/mL VEGF165 (sigma), 10 µM SB431542 and 2 µM forskolin. These endothelial cells were characterized for the expression of CD31 using flowcytometry and immunocytochemistry and were further evaluated for in vitro angiogenesis potential. These endothelial cells were found suitable for modeling vascularization of patient-derived organoids, gene expression analysis, and other downstream assays, that were performed.

### In vitro neural progenitor cells (NPCs) differentiation of human iPSCs

When human iPSCs cultured in mTeSR™1 media reached a confluency level of approximately 80%, they were passaged with StemPro® Accutase® Cell Dissociation Reagent (Thermo Fisher Scientific, Waltham, MA, USA) and resuspended as single cells in mTeSR™1 medium. Approximately 3 × 10^5^ cells/cm^2^ were seeded in six-well plates pre-coated with laminin and polyornithine diluted in DMEM/F12 (Thermo Fisher Scientific) for at least 1 h at 37 °C in a CO2 incubator. For initial differentiation, DMEM/F12 medium supplemented with 0.5% N2 supplement (GIBCO), 1 mM l-glutamine, 1% nonessential amino acids, noggin (500 ng/ml), SB431542 (10 µM) and laminin (1 µg/ml) was used.

For generation of NPCs, media was changed to neural induction medium (d7–14), containing DMEM/F12, 1% N2 supplement, 2% B27 supplement (Thermo Fisher Scientific), 1 µg/ml laminin, 20 ng/ ml basic fibroblast growth factor (stem cell technologies, USA). Once the cells are confluent (~ 80%), they were passaged with StemPro® Accutase® Cell Dissociation Reagent (Thermo Fisher Scientific, Waltham, MA, USA) and reseeded on six-well plates pre-coated with laminin and polyornithine diluted in DMEM/F12 (Thermo Fisher Scientific). Further NPCs were expanded in Neural progenitor expansion media containing DMEM/F12, 1% N2 supplement, 2% B27 supplement (Thermo Fisher Scientific), 1 µg/ml laminin, 20 ng/ml basic fibroblast growth factor, 20 ng/ml epidermal Growth Factor (stem cell technologies, USA).

On Day15, cells were considered pre-NPCs (passage 1) and able to be passaged (1:4) and cryopreserved when confluent. From passage 5, cells were considered NPCs and further used for neural differentiation studies. These NPCs were characterized by expression of Pax6, N-Cad and Nestin using flowcytometry and immunocytochemistry and utilized further for differentiation into mature neural derivatives (e.g. Midbrain, Forebrain Neurons, Astrocytes etc.), gene expression analysis, subjected to drug screening, metabolic analysis, and other downstream assays.

### In vitro forebrain motor neuron differentiation of human NPCs

NPCs were differentiated towards forebrain motor neurons by seeding dissociated single cells at 80–125,000 cells/cm^2^ density on polyornithine-laminin coated plates in advanced DMEM/F-12, 2% B27 supplement, 200 µM Ascorbic acid (Sigma), 0.65 µM Purmorphamine (Stem Cell Technologies) and 200 µM Dibutyryl cyclic-AMP (Sigma)) with 20 µM DAPT (γ-secretase inhibitor; Sigma) for 5–6 days. On day 7, cells were passaged with accutase (Stem cell technologies) and seeded at a density of 1.5 × 10^4^—3 × 10^4^ cells/cm^2^ in Advanced DMEM/F-12, 2% B27 supplement, 200 µM Ascorbic acid (Sigma), 0.65 µM Purmorphamine (Stem Cell Technologies) and 200 µM Dibutyryl cyclic-AMP (Sigma), DAPT was removed for next 5–6 days. The medium was changed every 3 days. These Forebrain motor neurons were characterized for the expression of Tuj1 using flowcytometry and immunocytochemistry and were utilized further for gene expression analysis, studying neurogenesis, neurodegenerative diseases, neuroinflammation and CNS function, subjected to neurotoxicity tests, drug screening, disease modeling, metabolic analysis and other downstream assays. Neurite outgrowth assay was performed to determine neurotoxicity. This assay offers a practical in vitro method for evaluating substances that inhibit or promote neurons' normal neurite development. We tested neurotoxic chemotherapeutic drug on iPSC-derived neurons- control (healthy donor), breast cancer and ovarian cancer patients [46–48] using ImageXpress® Nano Automated Imaging System (Molecular Devices).

### In vitro midbrain dopaminergic neuron differentiation of human NPCs

NPCs were differentiated to midbrain dopaminergic neurons by seeding dissociated single cells at 80—125,000 cells/cm^2^ density on polyornithine-laminin coated plates in DMEM/F12 medium supplemented with N2/B27/Glutamax (Invitrogen) containing retinoic acid (RA), ascorbic acid (AA), Sonic hedgehog (SHH), and FGF8 for 5–6 days. When cells reached 80—90% confluence, they were passaged with accutase (Stem cell technologies) and seeded at a density of 4 × 10^4^ to 6 × 10^4^ cells/cm^2^ in DMEM/F12 medium supplemented with N2/B27/Glutamax (Invitrogen) containing retinoic acid (RA), ascorbic acid (AA), and FGF8 for next 5–6 days. These Midbrain Dopaminergic Neurons were characterized for the expression of TUJ1 and Tyrosine Hydroxylase (TH) using flowcytometry and immunocytochemistry and further utilized for gene expression analysis, studying neurogenesis, neurodegenerative diseases, neuroinflammation and CNS function, subjected to neurotoxicity tests, drug screening, disease modeling, metabolic analysis, and other downstream assays.

### In vitro astrocyte differentiation of human NPCs

NPCs were differentiated to astrocytes by seeding dissociated single cells at 4 × 10^4^—6 × 10^4^ cells/cm^2^ density on matrigel-coated plates in DMEM/F12 medium supplemented with N2 supplement/B27/Glutamax (Invitrogen) containing BDNF (20 ng/ml, Peprotech), GDNF (10 ng/ml, PeproTech), Dibutyryl cyclic-AMP (250 µg/ml, Sigma), and L-ascorbic acid (200 nM Sigma); Ara-C (2 µg/l, Sigma), EGF (10 ng/ml, Sigma), LIF (10 ng/ml, Sigma), and FGF2 (10 ng/ml, Sigma) for 5–6 days. When cells reached 80—90% confluence, they were passaged with accutase (Stem cell technologies) and seeded at a density of 4 × 10^4^—6 × 10^4^ cells/cm^2^ in DMEM/F12 medium supplemented with N2 supplement/B27/Glutamax (Invitrogen) containing BDNF (20 ng/ml, Peprotech), GDNF (10 ng/ml, PeproTech), Dibutyryl cyclic-AMP (250 µg/ml, Sigma), and L-ascorbic acid (200 nM Sigma); Ara-C (2 µg/l, Sigma), EGF (10 ng/ml, Sigma), LIF (10 ng/ml, Sigma), and FGF2 (10 ng/ml, Sigma) + CNTF (20 ng/mL) for next 5–6 days. From D29 to D42, medium was changed every other day and cells were passaged once confluent. These astrocytes were characterized by expression of Tuj1 and GFAP or S100ß using flowcytometry and immunocytochemistry and utilized further for gene expression analysis, studying neurogenesis, neurodegenerative diseases, neuroinflammation and CNS function, subjected to neurotoxicity tests, drug screening, disease modeling, metabolic analysis, and other downstream assays.

### DNA and RNA extraction and PCR

gDNA and RNA was extracted from cells and organoids using the QIAamp® DNA Mini Kit (Qiagen, Venlo, Netherlands) and RNeasy Kit (Qiagen, Hilden, Germany) respectively as per manufacturer’s instruction. cDNA was synthesized using iScript cDNA synthesis kit (Biorad, California, United States). PCR was performed by using PCR Master Mix (2X) (ThermoFisher Scientific, Massachusetts, United States). The primer sequences used in PCR are provided as Supplementary Data (Table 1C).

### Immunofluorescence (IF)

iPSCs and derivatives were cultured on glass chamber slides (Thermo Scientific™ Nunc™ Lab-Tek™ II Chamber Slide™) for IF analysis. Cells were fixed with 4% Paraformaldehyde for 10 min at room temperature, permeabilized in 0.2%Triton™-X-100 (Sigma) for 10 min, blocked in 10% Bovine Serum Albumin (Life Technologies Inc.) for 60 min. Cells were then incubated with primary antibodies overnight (Supplementary Table 1A)., followed by secondary antibodies for 2 h at room temperature (Supplementary Table 1B). Subsequently, nuclei were stained with DAPI (Life Technologies Inc.) and images were captured with Evos FL Microscope (ThermoFisher).

### Immunohistochemistry (IHC) of tissues and organoids

FFPE blocks were prepared and sections were cut to a thickness of 3 µm. Deparaffinized, rehydrated, and antigen retrieved with citrate buffer (pH 6.0). The slides were blocked in 0.1% bovine serum albumin (BSA), 0.2% Triton X-100, and 0.05% Tween 20 in PBS for 1 h at room temperature (RT). The slides were then incubated overnight with primary antibodies against targets of interest (Supplementary Table 1A) in blocking buffer at 4^0^C. After washing, slides were incubated with secondary antibody (Supplementary Table 1B) for 45 min at 37^0^ C. Nuclei were counterstained with DAPI (Sigma). Images were acquired on the ImageXpress® Nano Automated Imaging System (Molecular devices, USA).

### Flow cytometry

Samples were prepared for flow cytometery analysis and 10,000 events were acquired using BD Accuri™ C6 Plus Flow Cytometer, (BD Biosciences, California, USA) and analyzed with FlowJo (FlowJo, RRID: SCR_008520) software.

### Cell viability assay

Cells were seeded at a density of 2500–5000 per well in 96-well clear bottom microplates. Cells were incubated overnight and treated with drugs for 3 days. Cell viability was analyzed using CellTiter-Glo (Promega, Wisconsin, USA) in SpectraMax ID5 Multimode Plate reader (Molecular devices, USA). IC50 values were calculated using GraphPad Prism version 5. Drugs used in the assays were purchased from Selleckchem (Texas, USA).

### Cell proliferation assay

The growth rate of organoids was measured. Briefly, the organoids were dissociated into single cells and 100,000 cells as initial setting were seeded into a 48-well plate, in triplicate. Cells were encapsulated in the Matrigel, were cultured in respective culture media for 8 days, then newly grown organoids were digested into single cells again, and the number of cells was counted with a haemocytometer and trypan blue exclusion method. The growth rate was calculated from the mean of three replicates using the following equation:

$$y\left(t\right)={y}_{0}\times {e}^{\left(\mathrm{growth\;rate}\;\times\;t\right)}$$

where y(t) is the number of cells at the final time point, y_0_ is the number of cells at the initial time point, and t is the time.

### In vitro PDO drug response testing

Patient-derived tumor organoid was dissociated to single cells after seven days in culture. Epithelial Cells (from tumor and adjacent normal tissues) in combination with patient-derived isogenic adipose-derived Mesenchymal Stem Cells: iECs: MSCs (3:1:1) were seeded at a density of 5000 cells per well in 96-well clear bottom microplates. Multicellular organoids were allowed to grow for seven days. Breast and ovarian organoids were imaged every day and fed every alternate day with aforementioned Breast organoid medium and Ovarian organoid medium respectively. After seven days in culture, feeding medium was replaced with feeding medium containing drug (-serum/FBS) at a concentration ratio of 1:1 ranging from 0.0016 × *C*_max_ to 125 × *C*_max_. Organoids were subsequently incubated for 0, 1, 7, 14 and 28 days; DMSO only- vehicle control and media only- negative control was used to set the baseline [49]. After 0, 1, 7, 14 and 21 days ATP levels were measured with the CellTiter-Glo (Promega, Wisconsin, USA). All screenings were performed in triplicates. Data were analyzed using GraphPad Prism 6. Drug dose–response curves were visualized using linear regression analysis [setting: log (inhibitor) versus response, least-squares (ordinary) fit; top constraint 100%]. Concentrations where 50% cell viability (IC50-value) was reached were interpolated. The area under the curve (AUC) was approximated between the lowest and highest concentrations screened in the actual assay with the trapezoid rule for numerical integration. The dose–response curves were plotted as the percentage of the cell viability against the logarithm of drug concentrations in µM and were fitted to estimate the IC50.

### Mycoplasma and Bioburden test detection

Absence of mycoplasma test performed routinely by MycoAlert® Mycoplasma Detection Kit as per manufacturer’s instruction. Bioburden testing performed routinely by plating spent media on Nutrient Agar Plate- for bacterial count (Merck) and on Sabouraud Agar Plate- for fungal count (Merck).

### Statistical analysis

Descriptive statistics including mean, SD and SEM were conducted with GraphPad Prism version 9.3.0 (GraphPad Prism, RRID: SCR_002798). Significance is represented by: *p < 0.5, **P < 0.01, ***p < 0.001, ****p < 0. 0001.The significance level for 95% confidence interval was set to = 0.05. The Pearson correlation test was applied to evaluate the correlation between replicate experiments. Curve-fitting algorithms for modelling drug response was also used for analysis and multivariate data was subjected to Bonferonni correction.

## Results

### Healthy donor and patient-derived PBMCs developed iPSC clones that express key pluripotency markers

**Fig. 1:**
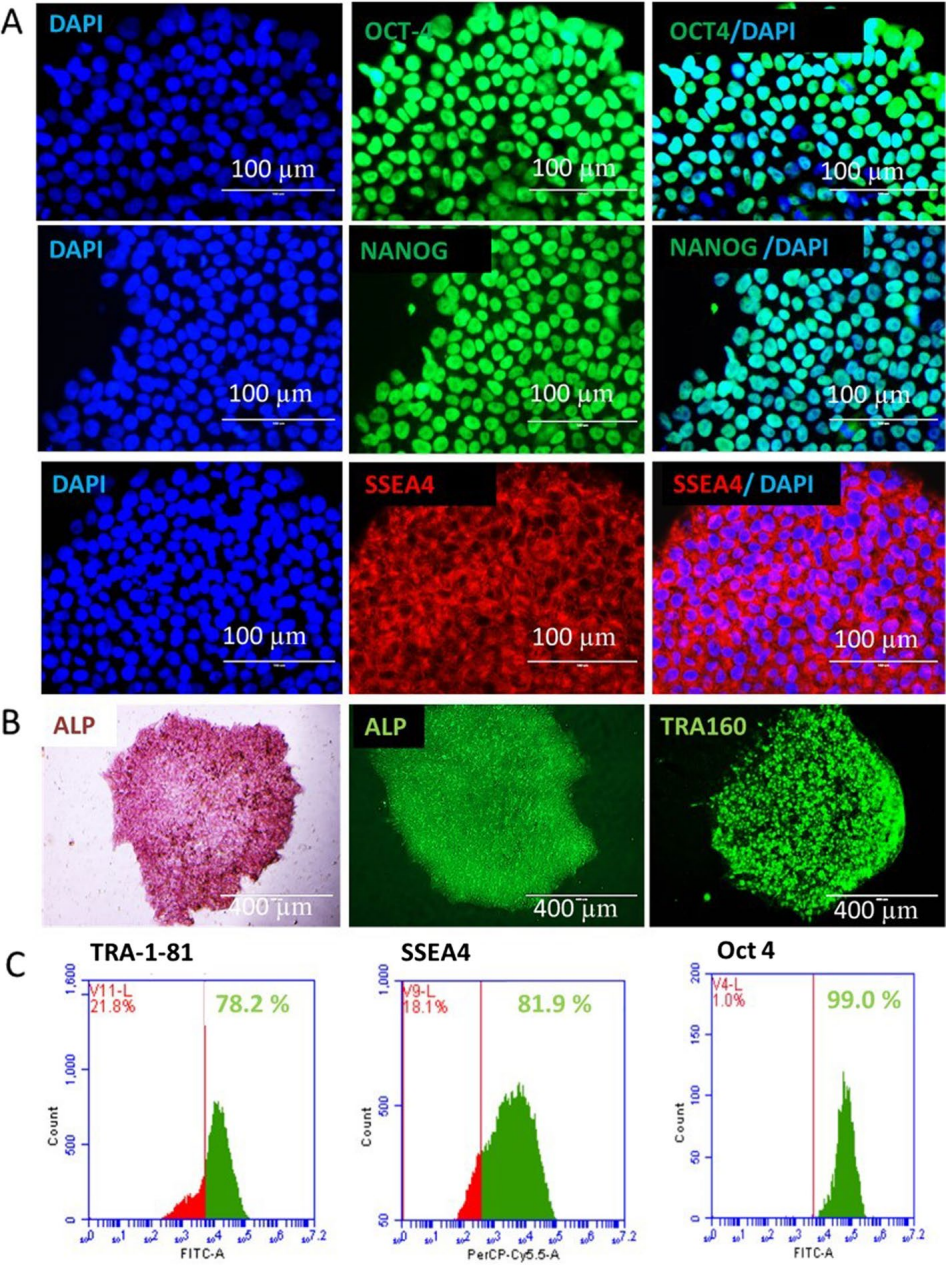
Representative images of **A**. Immunofluorescent analysis of iPSCs (healthy, breast cancer and ovarian cancer donors) for the pluripotency markers OCT4 (green), Nanog (green) and SSEA-4(red) with DAPI (Blue); Scale bar: 100 µm **B**. iPSCs positively stained with alkaline phosphatase (ALP) (chemical-pink & fluorescence-green) and TRA-1–60 (live image). Scale bar: 100 µm. Scale bar: 400 µm **C**. Flowcytometry analysis showing more than 70% expression of pluripotent markers TRA-1–81, SSEA4 and OCT4 (*green*) in iPSC lines

To generate human iPSCs from healthy donor, breast and ovarian cancer patients, while minimizing the risk of genomic abnormalities, we introduced the OSKM factors using the non-integrating sendai virus based interim gene modification technology. Colonies with a typical human ESC-like appearance began to emerge in culture 22 days after reprogramming. We observed that hiPSCs maintained undifferentiated morphology with round and clear edges in control conditions. The iPSC clones stained strongly positive for Alkaline Phosphatase activity as a test for pluripotency, and this positivity was maintained after passaging. The clones expressed pluripotent markers NANOG, OCT-4 and SSEA4. These clones also expressed > 70% SSEA-4, TRA-1–81 and OCT4 in flow cytometry analysis (Fig. 1).

### Healthy donor and patient derived iPSCs formed teratoma i.e. differentiated in vivo

Teratoma formation is considered a hallmark property of iPSCs when they are transplanted into immunodeficient mice. In order to check the in vivo pluripotency of the iPSC lines, teratoma assay was performed. In all transplanted animals, tumors were observed 4–6 weeks after transplantation. By 8–10 weeks the tumors were larger than 1 cm^3^ (average tumor volume of 2.2 ± 0.4 cm^3^) and the animals were sacrificed as per international animal ethics and regulatory norms. The tissues of the sacrificed animals' peritoneum, liver, spleen, and lungs were examined. The three germ layer structures were randomly arranged within the teratomas (Supplementary Fig. 1). In contrast, none of the control animals (*n* = 10) transplanted with 1 × 10^6^ healthy donor, breast cancer and ovarian cancer patients’ PBMCs either in the presence or absence of ROCK inhibitor, developed tumors or teratoma, as expected. These results demonstrated that subcutaneous transplantation of 0.5 to 1 million undifferentiated human iPSCs, combined with matrigel, into NOD/SCID mice was highly efficient, leading to teratoma formation in 100% of the transplanted mice, suggesting confirmation of pluripotency of the iPSC lines.

### Karyotypic and STR studies indicate genomic stability in healthy and patient iPSCs

Karyotypic analysis revealed no genomic abnormalities in patients’ iPSCs i.e. during reprogramming, no genomic instability was introduced in patients’ iPSCs. Both source PBMCs and iPSCs had normal diploid 46 karyotype, without acquired detectable abnormalities. STR DNA profiling analysis showed the genotypes of iPSC lines 100% matched with source donor's PBMCs and also confirmed the purity of the cell lines population, indicating that there is no cross-contamination from any other cell line [39–41].

### Neural progenitor differentiation under defined conditions

**Fig. 2:**
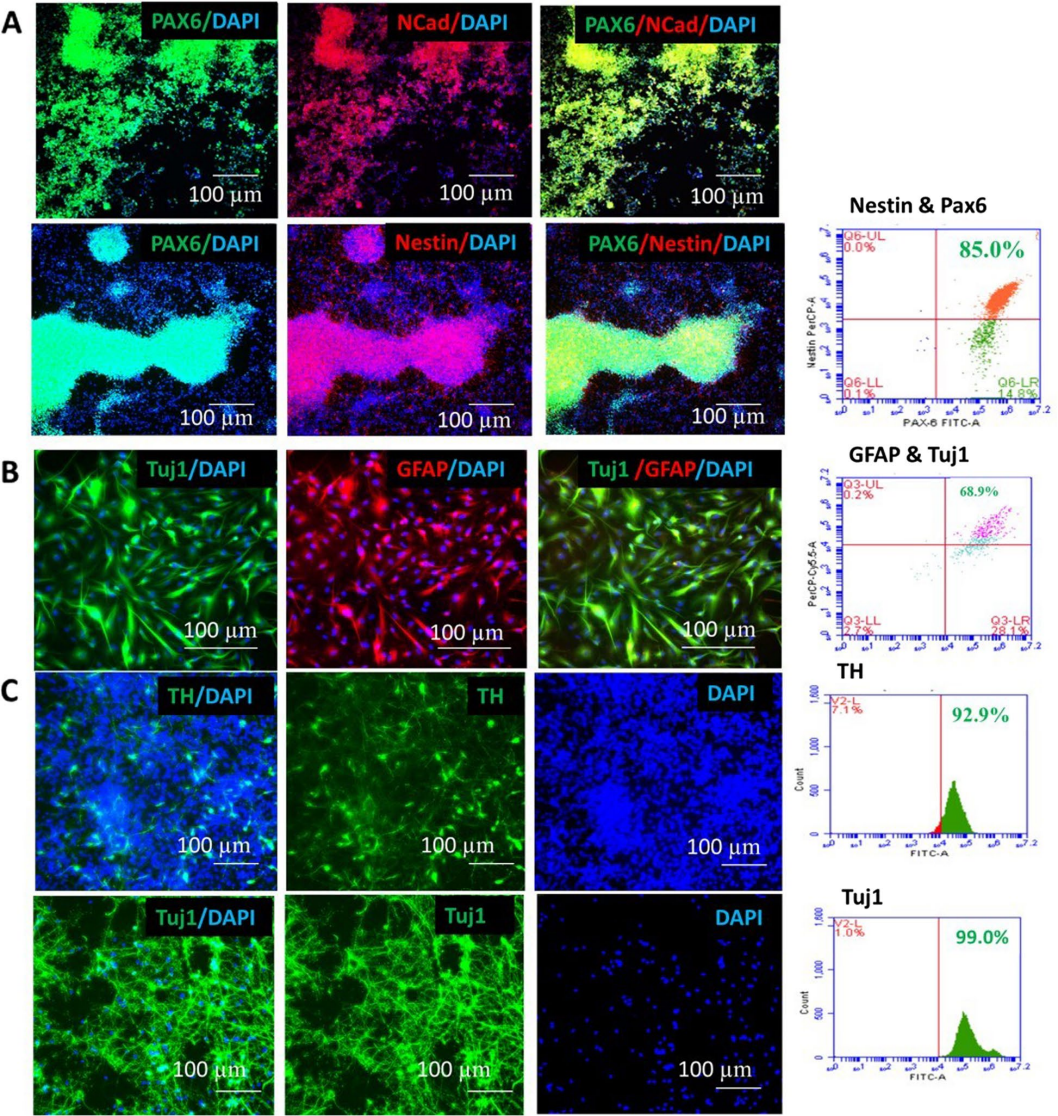
**A** Expression of neural progenitor cell markers Pax6 (green), NCad (red), Nestin (red) with nucleus (blue) in neural rosettes observed in neural progenitor cells. Flowcytometry analysis showed more than 80% expression of PAX6 and Nestin. Scale Bar:100 µm. **B** Expression of neural cell marker Tuj1 (green-ß tubulin) and astrocytes marker Glial fibrillary acidic protein (red-GFAP) in iPSCs-derived Astrocytes. Flowcytometry analysis showed more than 60% expression of Tuj1 and GFAP in iPSCs-derived Astrocytes. Scale bar: 100 µm. **C** Expression of neural cell marker Tuj1 (green-ß tubulin) and midbrain dopaminergic neural marker Tyrosine Hydroxylase (green-TH) in iPSCs-derived midbrain dopaminergic neurons with nucleus was observed. Flowcytometry analysis showed more than 80% expression of Tuj1 and TH in iPSCs-derived midbrain dopaminergic neurons. Scale bar: 100 µm

We differentiated hiPSCs from healthy donor, breast and ovarian cancer patients towards NPCs under defined, xeno-free conditions. This method yielded homogeneous and proliferative NPCs. Neural induction was assessed by expression of PAX6, an early marker of neuroectodermal development. Combined treatment with Noggin and SB431542 greatly increased the efficiency of neural induction where more than 80% of total cells were found PAX6 + . Results demonstrated that SB431542 and Noggin worked synergistically at several phases of differentiation to effectively transform hiPSC cells to neurons. Immunocytochemical analysis showed that, PAX6 + neuroectodermal cells express general neural stem cell markers, such as Nestin. More than 80% NPCs expressed PAX6 and Nestin (Fig. 2A).

### Forebrain motor neurons generated efficiently under xeno-free conditions

**Fig. 3:**
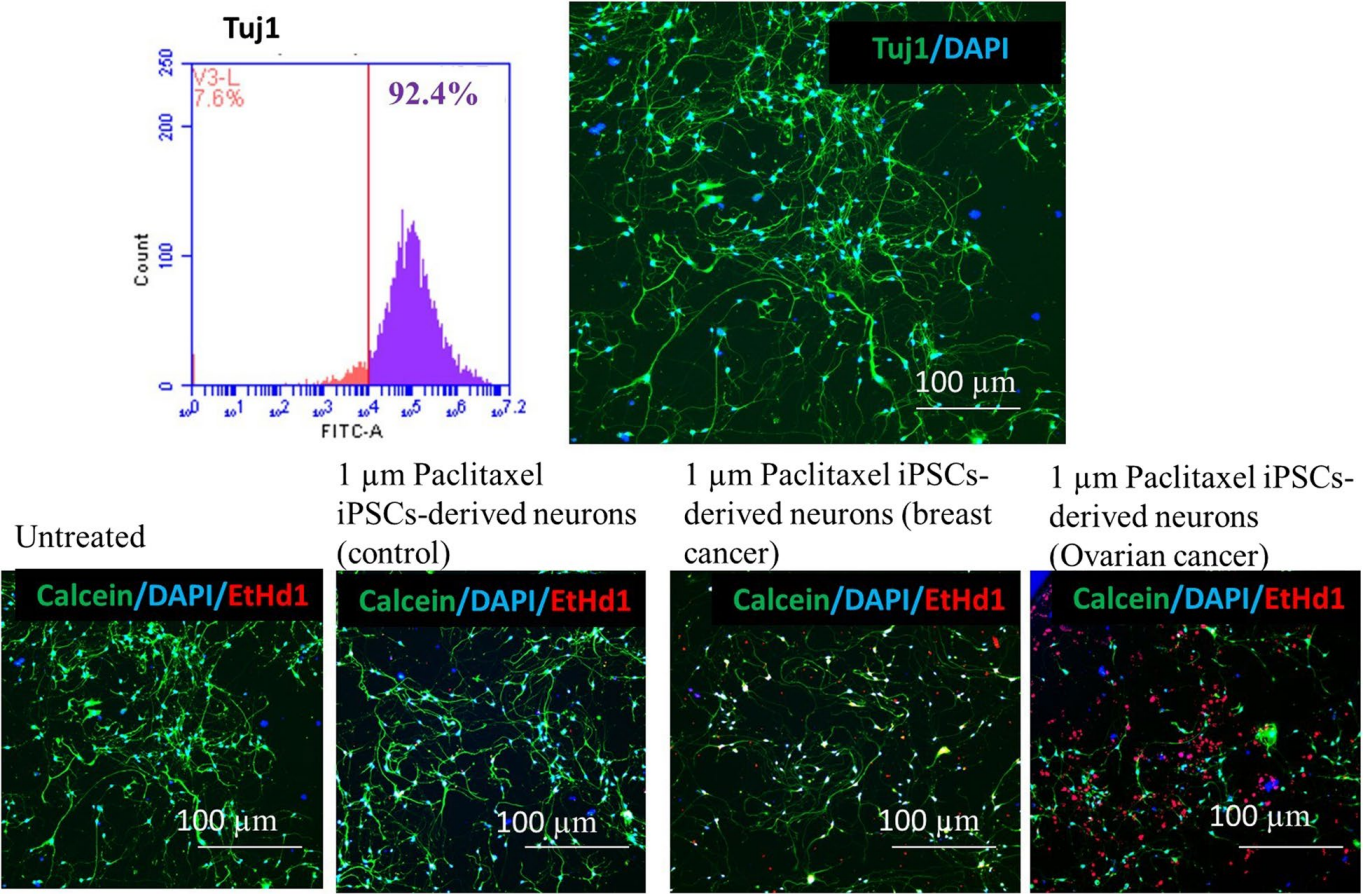
Flowcytometry analysis showed more than 80% expression of Tuj1 in iPSCs-derived forebrain motor neurons. Expression of neural cell markers Tuj1 (ß tubulin-green) was observed in iPSCs-derived forebrain motor neurons. Long neurites were visible with nuclei stained with DAPI. Neurite outgrowth sensitivity observed in iPSCs-derived neurons (control, breast cancer and ovarian cancer) using neurotoxic chemotherapeutic drug paclitaxel. Ovarian cancer and breast cancer neurons showed more toxicity than control. Scale bar: 100 µm

We further investigated whether hiPSCs had the capability to differentiate to forebrain motor neurons under xeno-free conditions. More than 90% of cells expressed neuronal marker ß III-tubulin at day 28, demonstrating that the differentiated neurons have a neuronal characteristic phenotype (Fig. 3). iPSCs-derived neurons from healthy donor, breast cancer and ovarian cancer patient were treated with 1 µM Paclitaxel and neurite outgrowth was analyzed. Ovarian cancer patient’s neurons were found more sensitive to paclitaxel followed by breast cancer patient and healthy donor (donor variability observed).

### Astrocytes were developed in a xeno-free environment

Astrocytes are critical components of the central nervous system. A number of mental and neurodegenerative disorders are linked to astrocyte dysfunction. To explore whether hiPSCs in xeno-free condition could differentiate to astrocytes, neural progenitors were treated with 20 ng/ml BDNF, 10 ng/ml GDNF, 10 ng/ml EGF, 250 µg/ml Dibutyryl cyclic-AMP, 10 ng/ml LIF, and 10 ng/ml FGF2 starting at d21^.^ With two months of continuous treatment, more than 60% of cells were GFAP & S100ß positive and ~ 30% were ßIII-tubulin positive (Fig. 2B).

### Midbrain dopaminergic neurons were generated under xeno-free condition

Neural patterning was initiated by a dual SMAD inhibition strategy [50] and specification towards a ventral midbrain dopaminergic neural fate was performed using the morphogens sonic hedgehog (SHH) and fibroblast growth factor-8a (FGF8a) [51]. It was observed that ascorbic acid, BDNF, GDNF and cAMP induced neural maturation. Extensive immunocytochemical analysis was performed on differentiated neurons and large populations (> 80%) of tyrosine hydroxylase- (TH) expressing cells were found (Fig. 2C).

### Cardiomyocytes were generated under xeno-free condition

**Fig. 4:**
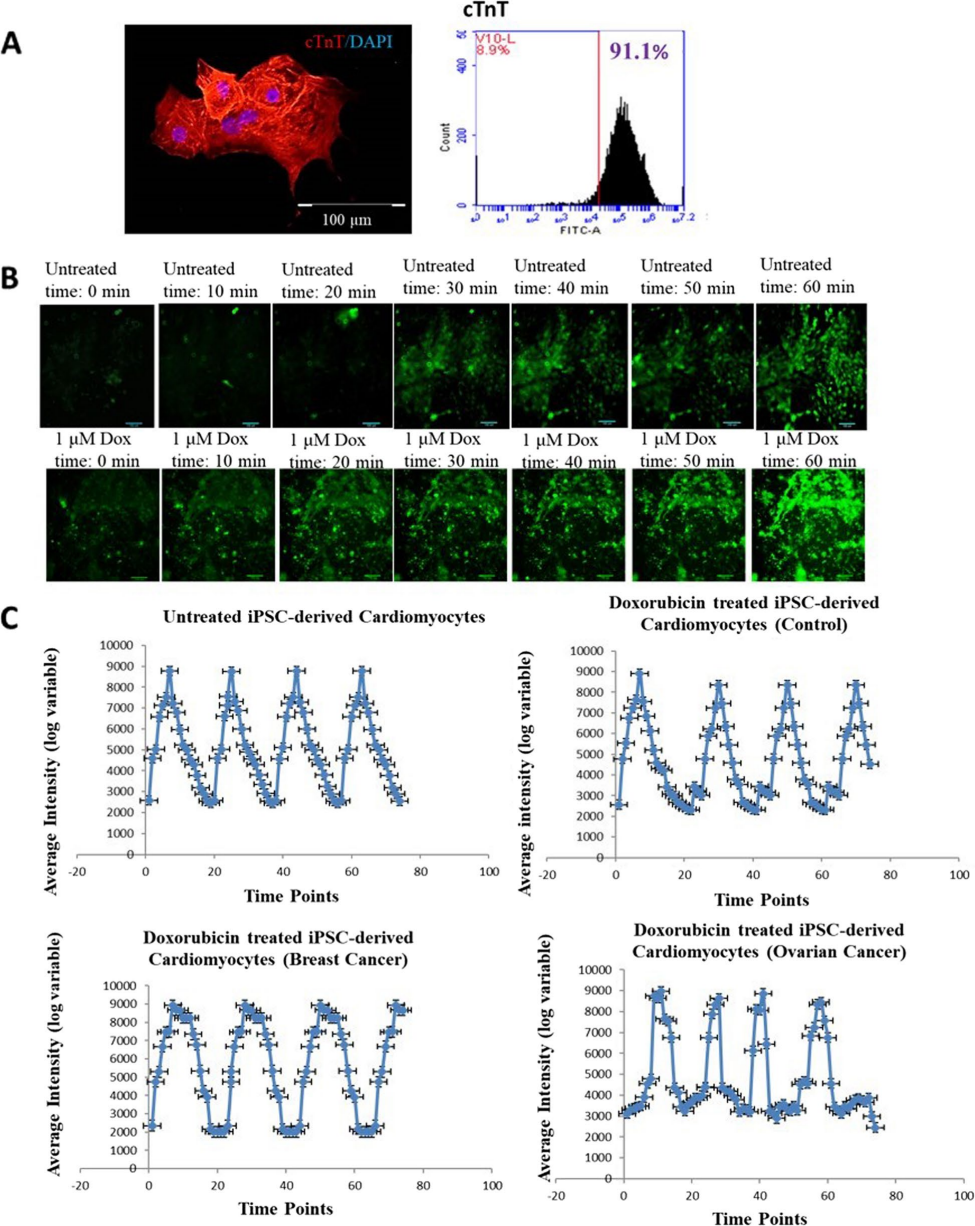
**A** Expression of cardiac marker cardiac troponin (green-cTnT) in iPSCs-derived cardiomyocytes with nucleus (blue) was observed. Flowcytometry analysis showed more than 80% expression of cardiac troponin in iPSCs-derived Cardiomyocytes. **B** Representative calcium-flux signal traces (average fluorescence intensities) for cardiotoxic compound-Doxorubicin. Traces shown are typical phenotypic responses including unaffected regular Ca^2^^+^ flux patterns, and affected doxorubicin treated iPSC-derived cardiomyocytes (Control, Ovarian cancer and Breast cancer) patterns, Scale bar: 100 µm. **C** Representative calcium-flux signal traces (average fluorescence intensities) for chemotherapeutic cardiotoxic drugs. Traces shown are typical phenotypic responses including untreated regular Ca^2+^ flux patterns, and treated doxorubicin patterns

60–70% confluency gave rise to a culture with the highest percentage of functional cardiomyocytes on day 15 of differentiation. On day 7, we observed small pockets of beating cardiomyocytes, and by day 8–10, a significant percentage of cells exhibited spontaneous contractions. To ensure that we were seeing appropriated maturation, we investigated the expression of mature cardiomyocyte markers such as cardiac troponin (cTnT) at a later stage of differentiation, D30. Flowcytometry analysis also revealed that more than 80% cells expressed cTnT (Fig. 4). iPSCs-derived cardiomyocytes from healthy donor, breast cancer and ovarian cancer patient were treated with 1 µM Doxorubicin (commonly used cardiotoxic chemotherapeutic drug) and calcium oscillation/contraction pattern was analyzed. Ovarian cancer patient’s cardiomyocytes was found more sensitive to Doxorubicin followed by breast cancer patient and healthy donor (donor variability observed).

### Differentiation of iPSCs to hepatocytes driven by growth factors

**Fig. 5:**
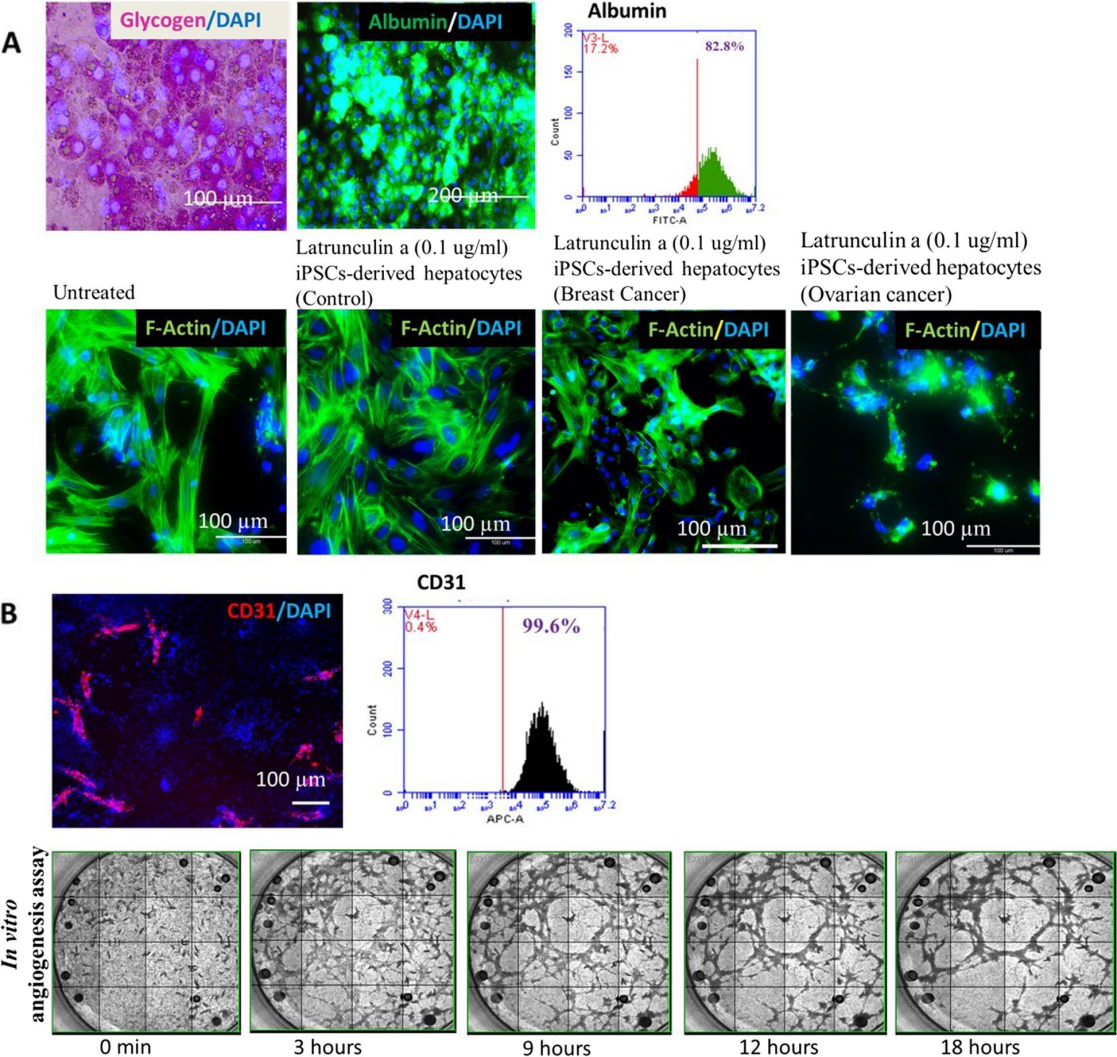
**A** Expression of glycogen storage (pink) and hepatic marker Albumin (green) in iPSCs-derived hepatocytes with nucleus (blue) was observed. Flowcytometry analysis showed more than 80% expression of Albumin in iPSCs-derived Hepatocytes. iPSC-derived hepatocytes (control, breast cancer and ovarian cancer patients) treated with Latrunculin showed sensitivity, Ovarian cancer and breast cancer hepatocytes showed more sensitivity than control. **B** Expression of endothelial marker CD31 (red-PECAM-1) in iPSCs-derived endothelial cells with nucleus (blue) was observed. Flowcytometry analysis showed more than 80% expression of CD31 in iPSCs-derived endothelial cells. Montage Image of in vitro angiogenesis assay on Matrigel revealed the potential to form capillary tubular networks of iPSC-ECs. Scale bar: 100 µm

A powerful and extremely specific GSK-3 inhibitor called CHIR99021 was applied to the cells for the first 24 h. After 24 h, the cells formed compact clusters, which were mesendodermal cells. At 48 h, the cells took on a pedal-like morphology. To specify a hepatic fate, the cells were treated with 1% DMSO for two days after which cells exhibited morphological change and were highly proliferative [42]. On day 7, cells exhibited typical hepatic progenitor morphology and were found positive for HNF4A and alpha fetoprotein. On day 15 of hepatic differentiation, primary hepatocyte-like cobblestone morphology was visible. These cells also demonstrated expression of albumin (Fig. 5A). In the final phase, cells were further treated with oncostatin and dexamethasone, a glucocorticoid mimetic, for hepatocyte maturation. On day 22, maturation was induced by oncostatin M and steroids like dexamethasone [42], cells exhibited complex cellular polarity and canaliculi network. More than 80% of cells expressed albumin as revealed by flowcytometry analysis (Fig. 5A). iPSCs-derived hepatocytes from healthy donors, breast cancer and ovarian cancer patients were treated with 0.1 ug/ml Latrunculin a (depolymerize actin filament) and analyzed for cytoskeleton disruption. Ovarian cancer patient’s hepatocytes were found more sensitive to Latrunculin followed by breast cancer patient and healthy donor (donor variability observed).

### Generation and characterization of endothelial cells derived from patients’ iPSCs

Using in vitro monolayer endothelial differentiation protocol, we successfully differentiated healthy donor, breast cancer and ovarian cancer patients’ iPSCs into endothelial cells (Fig. 5B). We observed endothelial cell-like i.e. cobblestone shaped elongated morphology on day 7 of endothelial induction. These cells also demonstrated expression of endothelial markers CD 31 in ICC staining (Fig. 5B). More than 80% of cells expressed CD31 as analyzed by flowcytometry (Fig. 5B). iPSCs-derived CD31 positive endothelial cells demonstrated potential of in vitro angiogenesis i.e. formation of vessel-like networks on matrigel (Fig. 5B).

### Establishment of primary ovarian cancer organoids

**Fig. 6:**
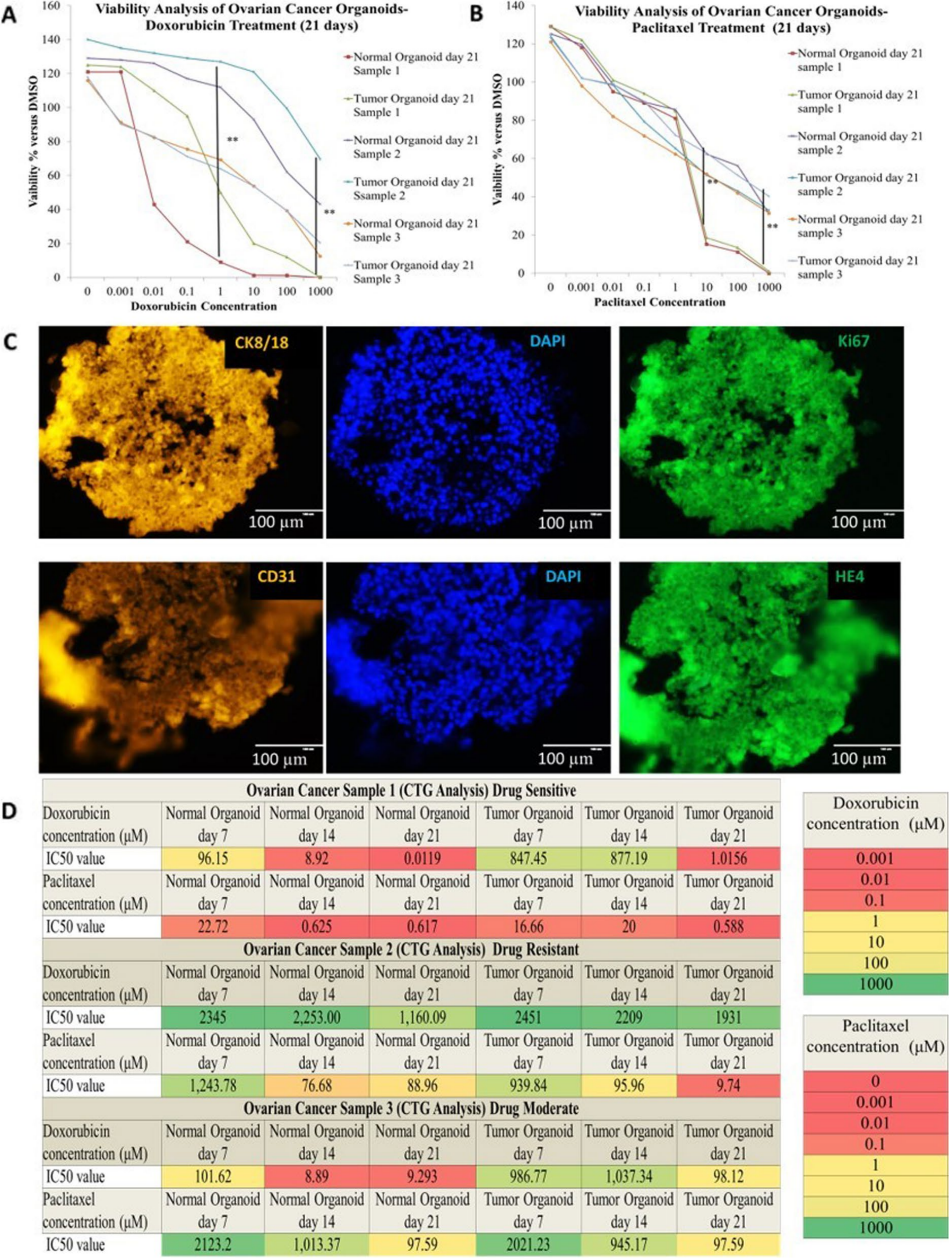
**A**, **B** Dose–response curves of the organoid lines treated with 2 FDA-approved compounds, paclitaxel and doxorubicin. Dots represent the mean of the technical duplicates. Error bars represent the SEM of technical duplicates. ** < *p* = 0.01, *N.S.*: not significant (one-way ANOVA). Data analyses were performed using the GraphPad Prism software. data are presented as mean ± SD). **C** Patient-derived primary ovarian cancer organoids maintain the histological architecture and expressed CK8/18, HE4, Ki67. Organoids also expressed CD31 of iPSCs-derived endothelial cells cocultured with patient derived epithelial cells to form organoids. Scale bar: 100 µm. **D** The heat map of sensitivity and resistivity of organoids against paclitaxel and doxorubicin, Summary of the 2 FDA-approved compounds used in the drug sensitivity and resistance testing (DSRT) and the results. The corresponding colors for IC50 are depicted in the legend

We developed a protocol to culture and expand organoids from tissues collected from ovarian cancer patients. We developed primary ovarian cancer organoids from various histologic subtypes (High grade serous carcinoma, moderately differentiated endometrial adenocarcinoma, and mucinous carcinoma) of stage I–III ovarian cancer patients in 2–3 weeks with matching adjacent normal tissue. The overall success of the primary organoid culture was ~ 80%. The developed organoids replicated the histological features of primary tumor. In less than three weeks, we developed expandable ovarian cancer organoids that accurately reflected the traits of many histological cancer subtypes. In terms of the expression of key molecular and cancer markers as well as therapeutic response, histological analysis of PDOs and the patient tissues from when they were initially produced revealed striking morphological parallel. We performed drug sensitivity and resistance test (DSRT) using 2 FDA-approved commonly used therapeutic drugs paclitaxel and doxorubicin. Depending on the properties of the individual drugs, the concentrations ranged from 0.001 µM to 1000 µM (Fig. 6).

### Establishment of primary breast cancer organoids

**Fig. 7:**
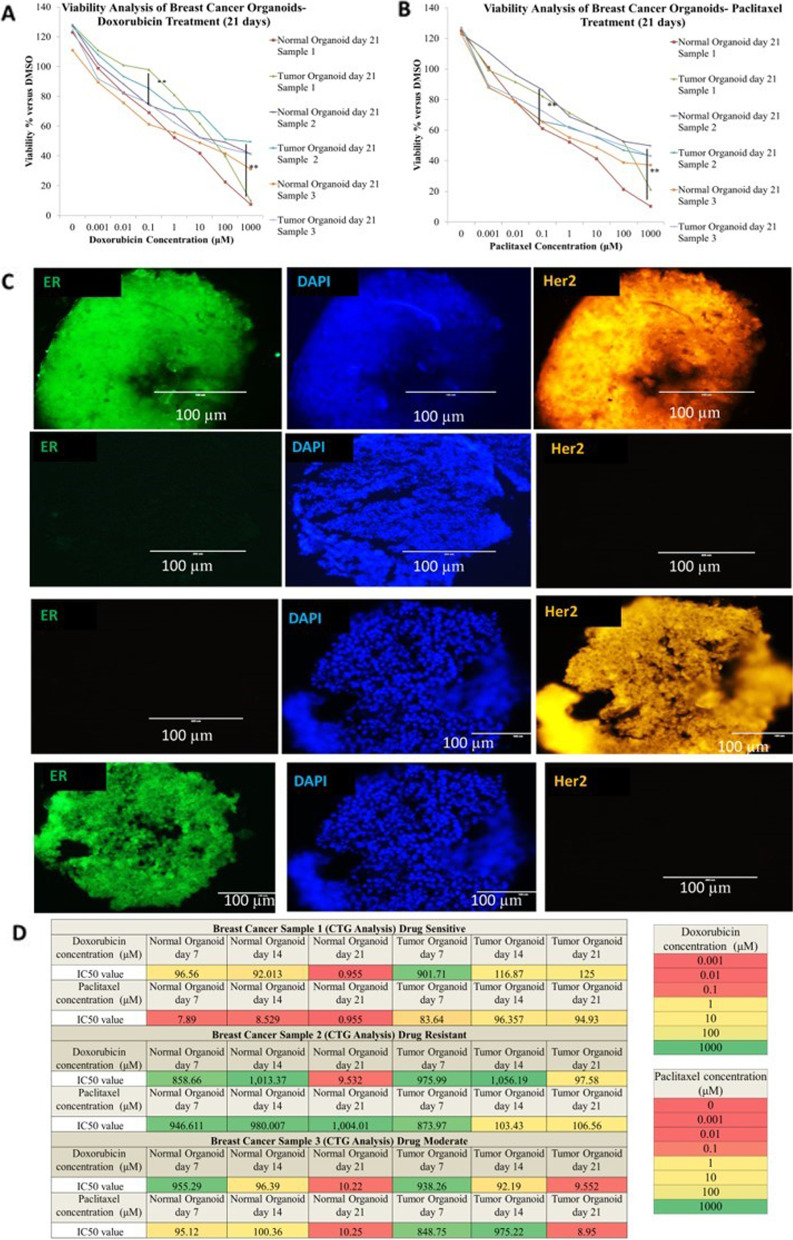
**A**, **B** Heterogeneous responses of organoids derived from breast cancer patients exposed to paclitaxel and doxorubicin. The fitted dose–response curves illustrate the responses of the organoids to paclitaxel and doxorubicin (*n* = 3 biologically independent data are presented as mean ± SD). **C** Organoids recapitulate the histological features of primary Breast Cancer Organoids. Immunohistochemical analyses show the examples of luminal A, luminal B, HER2-enriched and triple negative BCs, respectively. Scale bar = 100 µm. **D** The heat map of sensitivity and resistivity of organoids against paclitaxel and doxorubicin. Summary of the 2 FDA-approved compounds used in the drug sensitivity and resistance testing (DSRT) and the results. The corresponding colors for IC50 are depicted in the legend. Error bars represent the SEM of technical duplicates. ** < *p* = 0.01, *N.S.*: not significant (one-way ANOVA). Data analyses were performed using the GraphPad Prism software

We effectively produced matched organoid cultures using the normal and tumor tissues with a success rate of ~ 80% (12 out of 15) from human mammary tissues removed during mastectomies. Pathologists in each case validated the histology of the originating tissue. Viable BC organoids were obtained from luminal A, luminal B, human epidermal growth factor receptor 2 (HER2)-enriched and triple negative BC (TNBC; estrogen and progesterone receptors negative, HER2 negative). Organoids were produced from both invasive ductal carcinomas and invasive lobular carcinomas based on the histological characterization. Different samples of BC organoid cultures produced solid, cystic, cribriform, and "grape-like" structures that varied widely in size and morphology. PDO’s long term maintenance was higher for more aggressive tumor subtypes, TNBC and HER2-enriched PDOs having the highest proliferative potential and luminal A-derived PDOs having the lowest (Fig. 7).

Organoids were also developed from normal adjacent tissue (NAT) with glandular structures and mimicking mammary ducts. However, NAT-derived organoids propagated slowly, and lost proliferation after few passages. We investigated BC organoids in response to standard medications in order to assess their potential as in vitro disease models. Towards this, PDOs were treated with doxorubicin and paclitaxel (commonly used chemotherapeutic drugs). We found that few breast cancer PDOs were resistant to doxorubicin but were sensitive to treatment with paclitaxel.

### Organoids retained the genomic and histological features of the original tumor tissue

Histological evaluation revealed significant morphological similarities between PDOs and the patient tissues from which they were originally-derived. To compare the genomic characteristics of the parental tumor tissues and derived organoids, we did genomic DNA and cDNA analysis for selected ovarian cancer and breast cancer markers.

Tumor tissue derived organoids and snap frozen tissues were analyzed for *BRCA1, BRCA2, ER, PR and HER2* in breast cancer, *Tal2, EGF, ILF3, UBI2I, BRCA1* and *BRCA2* in ovarian cancer. Pluripotency markers *OCT4, SOX2* with cancer markers *BRCA1, BRCA2, ER, PR* and *HER2* in breast cancer iPSCs; *Tal2, EGF, ILF3, UBI2I, BRCA1* and *BRCA2* in ovarian cancer iPSCs were analyzed.

### Organoid usability for personalized Drug Sensitivity and Resistance Testing (DSRT)

Following seven days incubation, organoid cultures were manually inspected using a phase-contrast microscope to check cell health and morphology prior to the addition of drugs. CellTiter-Glo® 2.0 determines the number of viable cells in a well, based on the quantification of ATP present, an indicator of metabolically viable cells. Total cell count was measured by CellTiter-Glo® 2.0, which causes cell lysis and produces a luminescence signal proportional to the amount of ATP present [52]. Following completion of the above quality metrics, the data was normalized or the raw intensity data was used for curve-fitting.

- Data normalization: Normalization was completed using the following calculation: (Raw intensity signal- Mean of positive control/ Mean of negative control-Mean positive control)

For drugs where the concentrations selected have generated a dose–response curve, measurements such as the IC50 (half-maximal inhibitory concentrations) and AUC (area under the curve) were calculated to assess and compare sensitivity.

(b) Curve-fitting: Commercial software packages such as GraphPad Prism and Microsoft Office Excel were used to analyze the data generated from the drug screen assay. Curve-fitting algorithms for modeling drug response were also applied.

Finally, we performed DSRT using 2 FDA-approved drugs paclitaxel and doxorubicin. Ovarian cancer and breast cancer patient samples displayed heterogeneity in drug sensitivity pattern, on expected lines. Comparative studies were carried out across different patient samples and treatment groups. Notably, PDOs displayed simultaneous sensitivity or resistance to paclitaxel and doxorubicin. Organoids produced from metastatic cancers have a higher frequency of resistance to the microtubule- and nucleic acid synthesis-targeting drugs compared to primary malignancies.

We administered doxorubicin or paclitaxel to the resistant and sensitive organoid lines, to confirm the specific drug responses. We found apoptotic vesicles around the organoids in the sensitive lines after 24–72 h of treatment; these vesicles became more distinct after 72 h. Resistant organoids, however, retained distinct morphology until day 21 of drug treatment, as assessed by histological analysis.

## Discussion

Several ongoing global clinical trials are using organoids in correlation studies, including NCT03952793 in France, NCT04723316 in the UK, NCT02695459 in the Netherlands, NCT04927611 in China, NCT03896958 (Precision Insights on USA N-of-Effects in Georgia), central trial NCT02458716 at the Rutgers Cancer Institute of New Jersey in the USA and NCT03971812 in France.

Current clinical trials involve the use of iPSCs in modeling various disease types, include ophthalmologic, cardiovascular, and neurologic(clinicaltrials.gov, NCT03696628, NCT03971812, NCT02815072, NCT03853252). To our knowledge, our work presents first instance towards using an integrated patient-derived platform, that uses a holistic approach to check cardiotoxicity, hepatotoxicity, neurotoxicity as well as tumor and adjacent normal tissues-derived organoids-based drug screening to investigate safety and efficacy of drug(s) to help in precision medicine. In current study, we have developed human iPSCs from healthy donor, breast and ovarian cancer patients. Breast and Ovarian cancer patient tumor and normal adjacent tissues were used for development of patient organoids to test drug sensitivity and resistance. Patient’s adipose tissue-derived stromal cells/Mesenchymal Stem Cells were also used in development of organoids. iPSC-derived hepatocytes, cardiomyocytes, neurons and endothelial cells were also used for toxicity studies. During hepatotoxicity, cardiotoxicity and neurotoxicity studies, ovarian cancer derivatives showed more sensitivity towards drugs, followed by breast cancer and healthy donor (donor variability observed). Organoids were derived from both cancerous and normal adjacent tissues, originating from the same donor (isogenic samples). Thus, when organoids were exposed to drugs, they can help identify drugs efficacious against cancer cells while not compromising the safety of normal cells.iPSCs-derived hepatocytes present a valuable model that can closely resemble the phenotypes and functionality of primary hepatocytes while minimizing variability and other limitations of primary cells. In present study, we observed ovarian cancer patient’s iPSC derived hepatocytes to be more sensitive towards latrunculin followed by breast cancer and healthy donor, suggesting suitability of these hepatocytes for drug hepatotoxicity testing in preclinical studies. Similarly, human iPSC-derived cardiomyocytes not only closely resemble the phenotypes and functionality of primary cardiac cells but also avoid the sourcing and variability limitations of primary cells. In this study, we describe the development and optimization of methods to develop human iPSC-derived cardiomyocytes and illustrate how they can be used in phenotypic assays for cardiac toxicity assessment. It is reported that intracellular Ca^2+^ accumulation causes myocardial damage and reduces contractile performance [53]. In present study, we observed that calcium oscillations in ovarian cancer patients’ cardiomyocytes showed more sensitivity after Doxorubicin exposure, followed by breast cancer and healthy donor (donor variability observed). The higher degree of similarity with human cardiomyocytes compared to animal cardiomyocytes and potential for hiPSC-cardiomyocytes in high throughput screening for cardiac toxicity emerge as key benefits.

One of the most frequent adverse effects of cytotoxic chemotherapy is chemotherapy-induced peripheral neuropathy (CIPN), which is clinically distinguished by decreased sensation, sensory loss, and neuropathic pain. As model systems, rat pheochromocytoma or human neuroblastoma cell lines were used for in vitro investigations of CIPN to assess reductions in neurite outgrowth in response to neurotoxic chemotherapeutic agents such paclitaxel, vincristine, oxaliplatin, and cisplatin [54]. We developed an in vitro neurite outgrowth assessment using in house developed forebrain neurons using iPSCs from healthy donor, breast and ovarian cancer patients. In present study, we observed variable loss in the neurite process phenotypes upon paclitaxel treatment. Neurite outgrowth assessment showed paclitaxel sensitivity in ovarian cancer patients’ neurons followed by breast cancer and healthy donor (donor variability observed).

Our drug screening platform is a holistic approach which utilizes organoid cultures derived from tumor and isogenic histologically normal adjacent tissues that could be useful in identifying chemotherapeutic drug toxicity while the isogenic biorepository support biomarker discovery for predictive diagnostics and drug response. Understanding the genetic diversity of patients’ tumors and their influence on drug responses is an important step towards personalized medicine. In addition to research models for identifying biomarkers of response to new therapeutics or combinatorial regimens, we demonstrate that patient-derived organoids (PDOs) hold great promise as valuable preclinical models that can provide insight into case-specific drug responses.

Accumulating evidence suggests that PDOs can predict clinical outcomes in cancer patients [55–58]. Studies in several cancer types have shown that PDOs recapitulate both histological and genomic features of the lesion from which they were derived [58, 59]. In addition, PDOs can grow with high efficiency in a short period of time, which is much faster than generating a patient-derived xenograft (PDX models), allowing a priority prediction of response to chemotherapy with the potential to replace other regimens if primary resistance is established, thus improving patient survival [60]. PDOs can also be tested for response to new regimens including combinations of chemotherapy with targeted agents or multiple targeted agents that can be added to a patient's initial course of therapy  [61, 62].

Primary tumor PDOs considered highly responsive when the drug concentration that reduces viability by more than 50% of cells is lower than the concentration achievable in patient plasma (concentration steady state/maximum concentration Css/Cmax). However, the Css/Cmax vary between patients and is not necessarily the concentration that is achieved in the tumor [63]. We used the anthracycline chemical doxorubicin (dox) in the current study. It has cytotoxic properties, inhibits topoisomerase I, and efficiently prevents the synthesis of DNA and RNA in tumor cells. Doxorubicin was clinically approved by the Food and Drug Administration (FDA) in 1964 to treat a number of cancers, including ovarian cancer, thyroid cancer, stomach cancer, breast cancer, lymphoma, multiple myeloma, and sarcoma [64]. However, doxorubicin (Dox) is converted to semiquinone in the body, where it undergoes an oxidation event that might produce free radicals and damage the mitochondrial membrane of the myocardium. Myocardial toxicity happens when the myocardium's capacity to scavenge free radicals is low. The myocardium is more susceptible to Dox injury because of its substantially higher affinity for Dox than other human tissues. Acute or subacute damage that occurs right away after therapy or delayed cardiomyopathy that develops over time are both examples of cardiotoxicity. Studies reporting Cmax and AUC following a single administration at the highest dose suggested on the product label were chosen for this study because the mortality caused by Dox dose-dependent severe heart failure can be as high as 20%, which restricts its clinical application [65]. Typically, the maximum plasma concentration (Cmax) is expressed as ng/ml, and we converted this value into micromolar concentration units. As a crucial factor to take into account when transferring data from in vivo settings to in vitro systems with different protein compositions, the proportion bound to plasma protein is also included. Furthermore, the FDA "Orange Book" has mentioned Hepatotoxicity is a side effect of doxorubicin treatment and is mostly caused by the liver's role in the detoxifying process. Hepatocyte vacuolation, hepatocyte cord degeneration, bile duct hyperplasia, and localized necrosis are among the changes in the doxorubicin-treated liver tissue. By calculating the amounts of liver serum biomarkers, ROS generation, antioxidant enzymes, lipid peroxidation, and mitochondrial dysfunction, doxorubicin-induced hepatotoxicity has been described [66]. The liver blood biomarkers ALT and AST, elevated free radical levels causing oxidative stress described by an increase in Nrf-2, FOXO-1, and HO-1 genes, and a reduction in anti-oxidant activity characterized by a decrease in SOD, GPx, and CAT genes are all signs of oxidative stress. Increased values of SGOT, SGPT, LDH, creatinine kinase, direct, and total bilirubin further indicate that doxorubicin treatment caused toxicity in the hepatic tissue. The creation of ROS, reduced oxidative stress and inflammation, worsened mitochondrial synthesis and functioning, and increased apoptosis are the key molecular causes of hepatotoxicity. Dox is a regularly used anthracycline, and chemotherapy regimens including it are linked to cognitive decline and decreased neural connection in cancer survivors. Despite reports that Dox distribution to the central nervous system (CNS) is restricted, significant Dox concentrations have been seen in the brain when certain drugs are also administered. Pro-inflammatory cytokines can also weaken the blood–brain barrier because they are overproduced in cancer or in reaction to chemotherapy. Doxorubicin can cross blood–brain barrier through direct membrane-membrane connections with endothelial cells in some parts of the irregular endothelial basement membrane, and has a lot of vesicular activity.

In clinical practice, paclitaxel, a mitotic inhibitor, is frequently used as a first- or second-line anti-carcinogen for breast cancer and ovarian cancer. Inspite of significant clinical outcomes, paclitaxel's adverse effects, such as hepatotoxicity, cardiovascular toxicity, and neurotoxicity, must be taken into consideration. Paclitaxel causes peripheral neuropathy that is developed as a consequence of variable loss in the neurite process phenotypes upon increasing drug concentration. Paclitaxel and doxorubicin, anticancer medications that have been widely utilized in the treatment of breast, ovarian, certain head and neck, and lung cancers, were administered to the ovarian and breast cancer organoids. CTG assay confirmed that paclitaxel and doxorubicin treatments significantly decreased the viability of organoids with heterogeneity in drug resistance and sensitivity observed between cancer types in our study. The area under the concentration curve (AUC) for each replicate was calculated using the mean value in relation to control at each dose. AUCs were determined from 0.001–10 M or from 0.001–100 M for studies comparing the medicines, and from 0.001–100 M for analyses comparing the three iPSC-derivatives. One-way ANOVA was used to determine the significance of the variations in AUCs between the medications or between the biological replicates of iPSCs for each phenotype, without assuming equal variances. The conventional 2D cell culture system, which has been extensively employed in earlier studies, is inferior to the revolutionary 3D culture method, which mimics the rich in vivo milieu and intricate mechanisms through which cells grow, thus showing significant promise for the preclinical assessment of medication effectiveness and related risks.

Our study supports the use of PDOs to model different types of breast and ovarian cancers, to unveil intrinsic therapy-resistant sub-clones in heterogeneous carcinomas, and to explore new and/or alternate therapeutic strategies. We demonstrated that these models can be employed as in vitro platforms to test combination therapies for drug resistance as well as the sensitivity of cancer cells to conventional medicines. Our results support the usage of PDOs to capture different features of breast and ovarian cancer, but it also highlights the need to further develop this methodology with co-culturing tumor cells with immune cells in suitable matrices that imitate the physical characteristics of the complex tumor immune microenvironment (TIME) [67]. Cancer immunotherapy is a type of therapy that boosts the body’s own immune system to fight cancer. Due to the limitations of conventional in vivo animal models and 2D in vitro models, they fail to accurately replicate the intricate TIME of source tumor. In addition, due to the involvement of the immune system in cancer immunotherapy, more physiomimetic cancer models, such as PDOs, are required to evaluate the efficacy of immunotherapy agents [68].

## Conclusion

The pressing need to improve cancer immunotherapies has brought great attention to the tumor immune microenvironment (TIME), whose study requires robust and faithful preclinical models recapitulating patient-specific tumor-immune interactions.

We observed in many cancer patients, tumor was clinically resistant to doxorubicin and paclitaxel and in few cases found sensitive in normal adjacent tissue derived organoids. Therefore, our 3D patient-derived organoids model could be used to develop efficacious clinical regimes with minimal to no safety concerns in healthy tissues, while also facilitating vistas for combinatorial therapy.

Further we also developed isogenic Neural (Midbrain dopaminergic neurons, forebrain motor neurons and astrocytes), hepatic and cardiac cells from ovarian and breast cancer patients which have been used to study drug efficacy along with investigating neurotoxicity, hepatotoxicity and cardiotoxicity. Our pioneering integrated patient-derived organoid technology holds great promise for clinical advancements and personalized therapy to overcome the clinical challenges of heterogeneity and increasing cytotoxicity observed in patients, in response to cancer treatment.

### Supplementary Information

**Additional file 1.**

## Data Availability

All data needed to evaluate the conclusions in the paper are present in the paper and/or the supplementary material. Raw data will be available upon request (contact email: swati.chitrangi@yashraj.com).

## Abbreviations

iPSCs: Induced Pluripotent Stem Cells
PDO: Patient-derived Organoids
PDX: Patient-derived Xenograft
TIME: Tumor Immune Microenvironment
TME: Tumor Microenvironment
CTG: CellTiter-Glow
3D: Three Dimensional
IC-SCR: Institutional Committee for Stem Cell Research
NAC-SCRT: National Apex Committee for Stem Cell Research and Therapy
IEC: Institutional Ethics Committee
ICMR: Indian Council of Medical Research
DCGI: Drug Controller General of India
IAEC: Institutional Animal Ethics Committee
IBSC: Institutional Biosafety Committee
TDB: Technology Development Board (TDB),
DST: Department of Science and Technology
ECM: Extracellular Matrix
CAF: Cancer Associated Fibroblast
CDX: Cell-derived Xenograft
2D: Two Dimensional
MSC: Mesenchymal Stem Cells
HTS: High-Throughput Screening
PBMC: Peripheral Blood Mononuclear Cells
DMSO: Dimethyl Sulfoxide
FFPE: Formalin-Fixed Paraffin-Embedded
NBF: Neutral buffered formalin
CD: Clusters of Differentiation
BMP4: Bone morphogenetic protein 4
bFGF: Basic fibroblast growth factor
NPC: Neural progenitor cells
TH: Tyrosine Hydroxylase
SHH: Sonic Hedgehog
BDNF: Brain Derived Neurotrophic Factor
GDNF: Glial Cell Line-Derived Neurotrophic Factor
LIF: Leukemia inhibitory factor
EGF: Epidermal growth factor
FGF2: Fibroblast Growth Factor 2
CNTF: Ciliary Neurotrophic factor
CNS: Central Nervous System
GFAP: Glial Fibrillary Acidic Protein
IHC: Immunohistochemistry
iEC: iPSCs-derived Endothelial Cells
AUC: Area Under Curve
IC50: Half Maximal Inhibitory Concentration
SD: Standard of Deviation
SEM: Standard Error of Mean
cTnT: Cardiac Troponin
ME: Mesendoderm
DSRT: Drug Sensitivity and Resistance Test
FDA: Food and Drug Administration
Her2: Human Epidermal Growth Factor Receptor 2
NAT: Normal Tissue Adjacent to the Tumor
BC: Breast Cancer
CIPN: Chemotherapy Induced Peripheral Neuropathy
CSS: Concentration of drug in plasma at Steady State
Cmax: Drug to reach the Maximum Concentration
Tmax: Time to peak drug concentration
ROS: Reactive Oxygen Species
ALT: Alanine Transaminase
AST: Aspartate Aminotransferase
SGOT: Serum Glutamic Oxaloacetic Transaminase
SGPT: Serum Glutamic Pyruvic Transaminase
LDH: Lactate Dehydrogenase
ANOVA: Analysis of Variance
ICC: Immunocytochemistry
CPSEA: Committee for the Purpose of Control and Supervision of Experiments on Animals
