# Correction: Patient-derived organoids for precision oncology: a platform to facilitate clinical decision making

**DOI:** 10.1186/s12885-023-11269-4

**Published:** 2023-08-18

**Authors:** Swati Chitrangi, Pooja Vaity, Aishwarya Jamdar, Shweta Bhatt

**Affiliations:** 1 Department of Integrated Drug Discovery and Development, Yashraj Biotechnology Limited, C-232 and C-113, TTC Industrial Area, MIDC, Pawane, Maharashtra 400705 India; 2 Yashraj Biotechnology GmbH, Uhlandstraße 20-25, 10623 Berlin, Germany; 3 Yashraj Biotechnology Limited, 8, The Green STE A, Dover, Delaware State 19901 USA

**Correction: BMC Cancer 23, 689 (2023)**

10.1186/s12885-023-11078-9

Following publication of the original article [1], the authors reported that Figs. 4 and 5 were erroneously transposed. The original article [1] has been corrected.

**Fig. 4:**
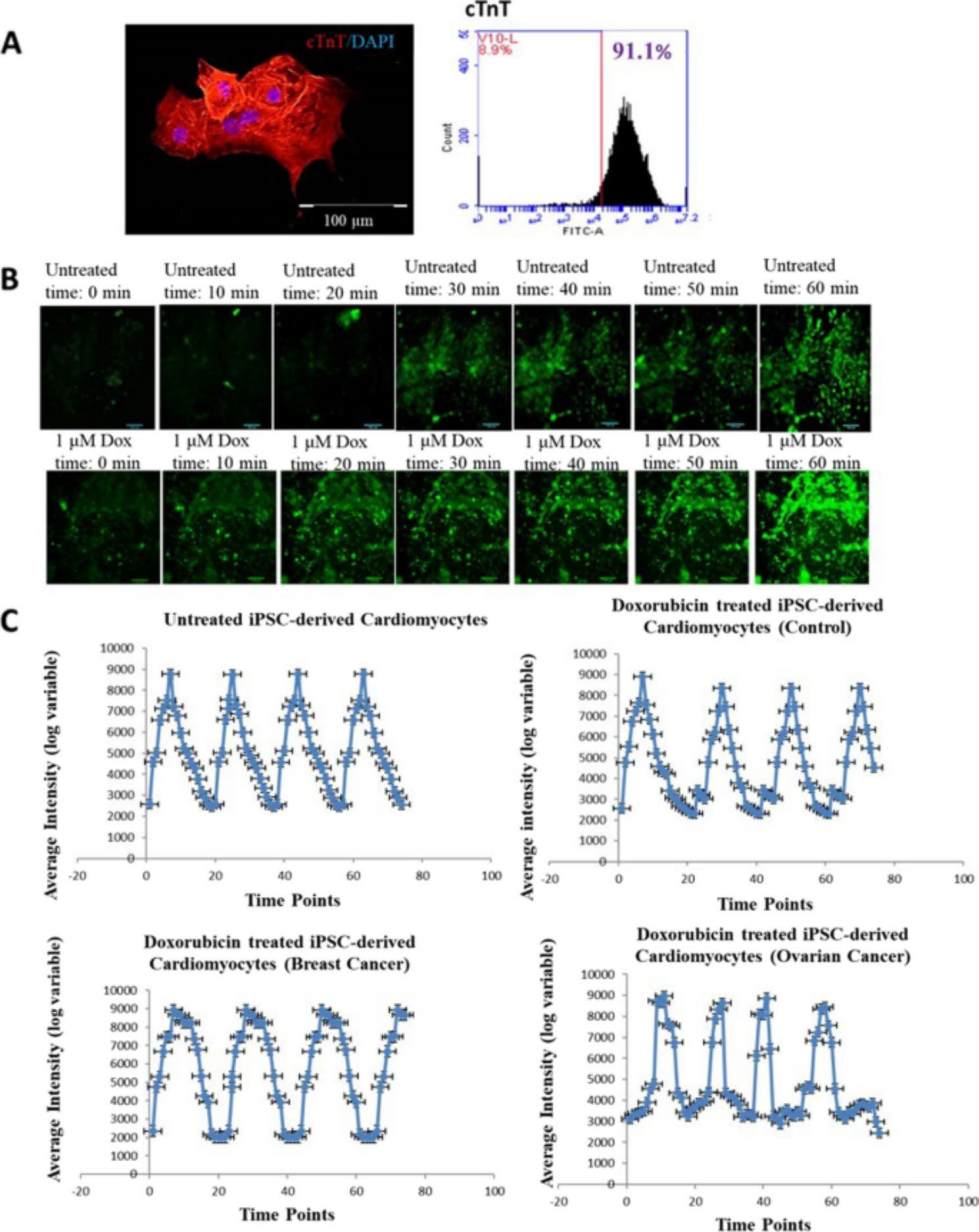
**A** Expression of cardiac marker cardiac troponin (green-cTnT) in iPSCs-derived cardiomyocytes with nucleus (blue) was observed. Flowcytometry analysis showed more than 80% expression of cardiac troponin in iPSCs-derived Cardiomyocytes. **B** Representative calcium-flux signal traces (average fluorescence intensities) for cardiotoxic compound-Doxorubicin. Traces shown are typical phenotypic responses including unaffected regular Ca^2+^ flux patterns, and affected doxorubicin treated iPSC-derived cardiomyocytes (Control, Ovarian cancer and Breast cancer) patterns, Scale bar: 100 μm. **C** Representative calcium-flux signal traces (average fluorescence intensities) for chemotherapeutic cardiotoxic drugs. Traces shown are typical phenotypic responses including untreated regular Ca^2+^ flux patterns, and treated doxorubicin patterns

**Fig. 5:**
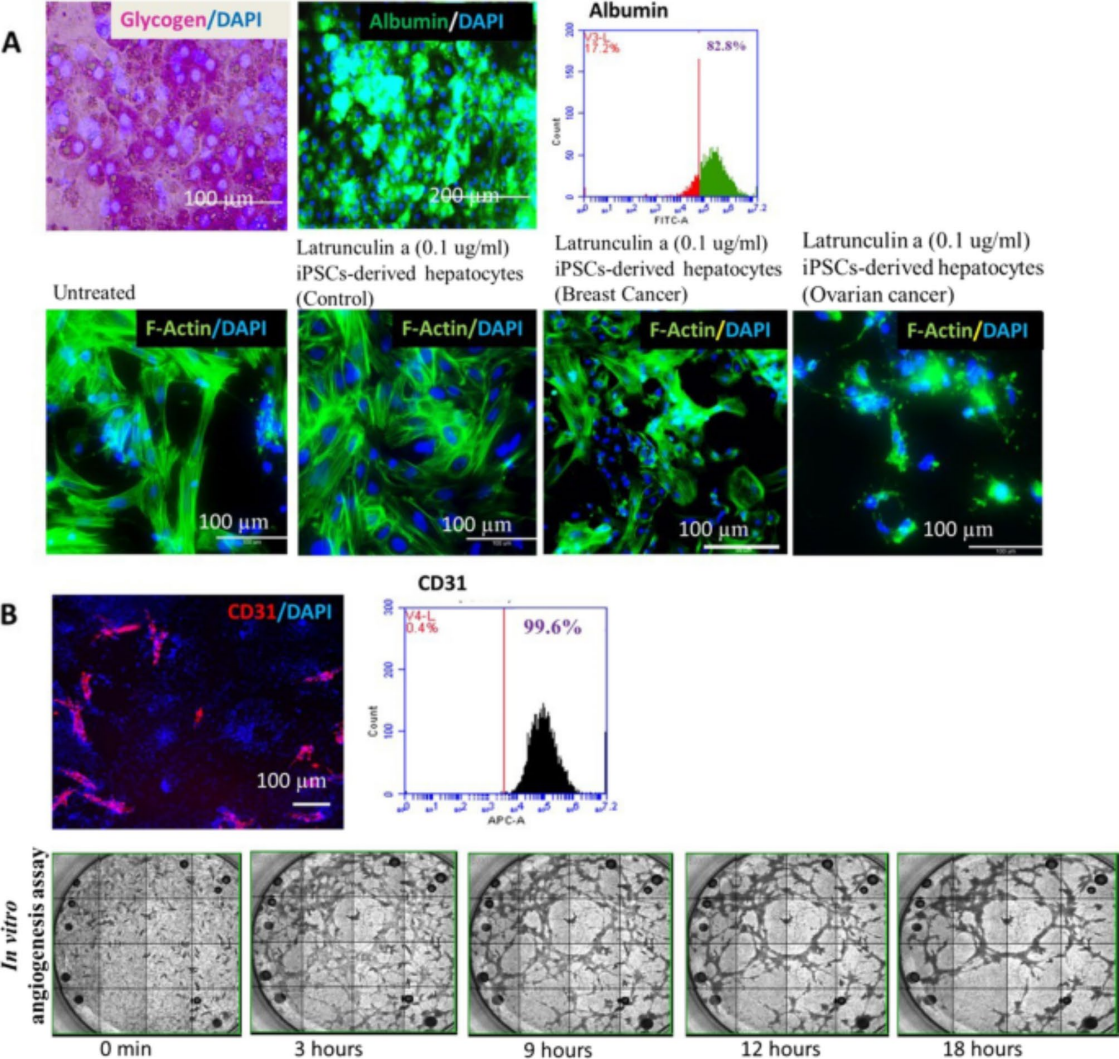
**A** Expression of glycogen storage (pink) and hepatic marker Albumin (green) in iPSCs-derived hepatocytes with nucleus (blue) was observed. Flowcytometry analysis showed more than 80% expression of Albumin in iPSCs-derived Hepatocytes. iPSC-derived hepatocytes (control, breast cancer and ovarian cancer patients) treated with Latrunculin showed sensitivity, Ovarian cancer and breast cancer hepatocytes showed more sensitivity than control. **B** Expression of endothelial marker CD31 (red-PECAM-1) in iPSCs-derived endothelial cells with nucleus (blue) was observed. Flowcytometry analysis showed more than 80% expression of CD31 in iPSCs-derived endothelial cells. Montage Image of in vitro angiogenesis assay on Matrigel revealed the potential to form capillary tubular networks of iPSC-ECs. Scale bar: 100 μm
