# Supplementary material for: Patient-derived organoids for precision oncology: a platform to facilitate clinical decision making
Source: BMC Cancer. 2023 Jul 22;23:689. doi: 10.1186/s12885-023-11078-9 (PMC10362580; doi:10.1186/s12885-023-11078-9)
Supplement: Supplementary file 1 — Additional file 1. [file 12885_2023_11078_MOESM1_ESM.docx]

**Supplementary files**


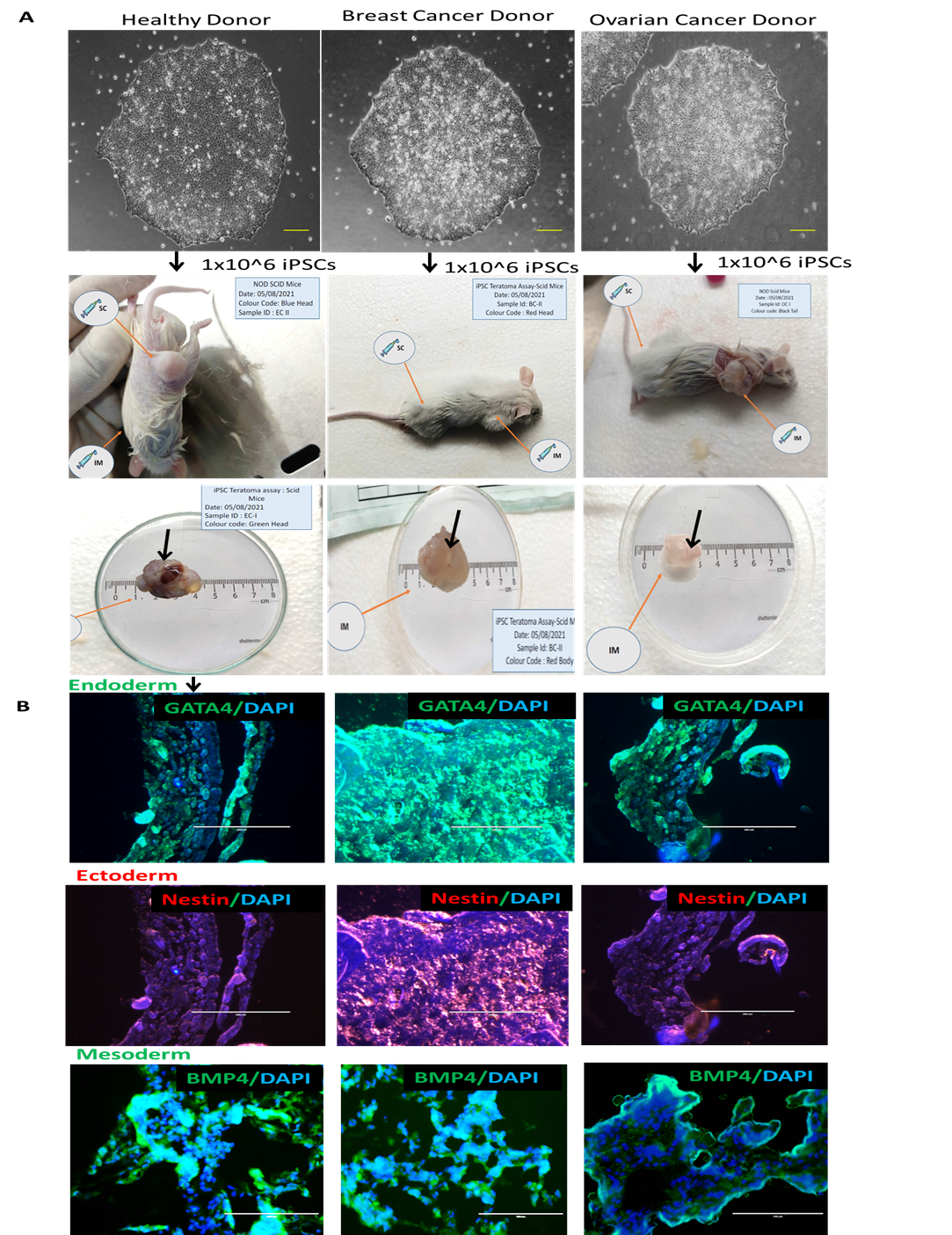


**Figure 1 (Supplementary)**: Representative microscopic images of A. iPSCs (healthy, breast cancer and ovarian cancer donors), showing teratoma formation in immunocompromised mice. B. Immunohistochemical analysis of teratoma showed expression of endoderm (GATA4), ectoderm (Nestin) and mesoderm (BMP4) markers.Scale bar: 200 µm

**Table 1A: Primary antibodies list:**

| **Sr. No.** | **Name of antibody** | **Host** | **Make** | **Catalog No.** | **Dilution** |
| --- | --- | --- | --- | --- | --- |
| **For Characterizing iPSCs** | | | | | |
| **1** | Anti-Alkaline Phosphatase | Mouse | R & D system | SC008 | 1:100 |
| **2** | Anti-Nanog | Goat |  |  | 1:100 |
| **3** | Anti-Oct-4 | Goat |  |  | 1:100 |
| **4** | Anti-SSEA-1 | Mouse |  |  | 1:100 |
| **5** | Anti-SSEA-4 | Mouse |  |  | 1:100 |
| **6** | N-Cadherin | Mouse | Sigma | C3865 | 1:100 |
| **7** | GATA-4 | Rabbit | Sigma | HPA073899 | 1:100 |
| **8** | BMP4 | Rabbit | Atlas Antibodies | HPA066235 | 1:100 |
| **9** | Tuj1 | Rabbit | Cell Signaling | 5568T | 1:100 |
| **10** | GFAP | Rabbit | Cell Signaling | 3670T | 1:100 |
| **11** | Tyrosine hydroxylase | Mouse | Abcam | Ab112 | 1:100 |

**Table 1B: Secondary antibody list:**

| **Sr. No.** | **Name of antibody** | **Host** | **Make** | **Catalog No.** | **Dilution** |
| --- | --- | --- | --- | --- | --- |
| **1** | Goat Anti-Mouse IgG H&L (Alexa Fluor 594) Preadsorbed | Goat | Abcam | ab150120 | 1:200 |
| **2** | Goat Anti-Rabbit IgG H&L (Alexa Fluor 488) Preadsorbed | Goat | Abcam | ab150081 | 1:200 |

**Table 1C: Primer list:**

| **Primers used for PCR** | | | |
| --- | --- | --- | --- |
|  | **Target** | **Forward Primer (5’-3’)**  **Reverse Primer (5’-3’)** | **Size of Band (bp)** |
| Housekeeping Genes | 18s rRNA | F: 5’-TCTGCTCTTCGCGTTGAAGAA-3’  R: 5’-CACTCTTGTGCTGACTTACCA-3’ | 171 |
| Trilineage Genes | Nestin | F: 5'-TCCAGAAACTCAAGCACCA-3'  R: 5'-AAATTCTCCAGGTTCCATGC-3' | 183 |
|  | PAX6 | F-5’GATAACATACCAAGCGTGTCATCAATA-3’  R-5’TGCGCCCATCTGTTGCT-3’ | 75 |
|  | N-CAD | F 5’- TCCTGATATATGCCCAAGACAA-3’  R 5’-TGACCCAGTCTCTCTTCTGC-3’ | 183 |
|  | GATA-4 | F: 5’-TAGACCGTGGGTTTTGCATTG-3’  R: 5’-CATCCAGGTACATGGCAAACAG-3’ | 119 |
|  | cTNT | F: 5’-AGCATCTATAACTTGGAGGCAGAG-3’  R: 5’- TGGAGACTTTCTGGTTATCGTTG-3’ | 111 |
|  | Albumin | F:5’-TGCTTGAATGTGCTGATGACAGGG-3’  R: 5’-AAGGCAAGTCAGCAGGCATCTCATC-3’ | 161 |
|  | Alpha-fetoprotein (AFP) | F: 5’-TGAAATGACTCCAGTAAACCC-3’  R: 5’-AATGAGAAACTCTTGCTTCATC-3’ | 199 |

**Table 2: Clinical sample details**

| **Sr. No.** | **Sample ID** | **Donor’s Gender**  **/Age** | **Donor’s Race** | **Morbidity** | **Cancer Stage** |
| --- | --- | --- | --- | --- | --- |
| 1 | YBL0001 | F/44 | Asian | Ca. Breast | Invasive duct carcinoma, grade III with metastatic regional nodes, pTNM - T2 N2 |
| 2 | YBL002 | F/69 | Asian | Ca. Ovary | Moderately differentiated papillary serous adenocarcinoma I involving right ovary infiltrating into myometrium and sigmoid colonic wall |
| 3 | YBL003 | F/45 | Asian | Ca. Ovary (Serous cystadenoma) | Metastatic poorly differentiated carcinoma |
| 4 | YBL004 | F/54 | Asian | Ca. Breast | Invasive duct carcinoma, grade III with metastatic regional nodes; pTNM - T2 N2 |
| 5 | YBL005 | F/36 | Asian | Ca. Breast | Invasive duct carcinoma, grade III with reactive regional nodes |
| 6 | YBL006 | F/68 | Asian | Ca. Breast | Infiltrating duct carcinoma, grade III with left axillary nodal metastasis |
| 7 | YBL007 | F/61 | Asian | Ca. Breast | Invasive duct carcinoma; grade III with reactive regional nodes; pTNM - T2 N0 |
| 8 | YBL008 | F/63 | Asian | Ca. Breast | Invasive ductal carcinoma, Grade II with metastatic regional nodes |
| 9 | YBL009 | F/65 | Asian | Ca. Ovary | Metastatic adenocarcinoma |
| 10 | YBL010 | F/51 | Asian | Ca. Breast | Invasive ductal carcinoma, grade III with metastatic regional nodes, pTNM - T2N1 |
| 11 | YBL011 | F/65 | Asian | Ca. Breast | Invasive ductal carcinoma, grade III with metastatic regional nodes, pTNM - T2N1 |
| 12 | YBL012 | F/41 | Asian | Ca. Breast | Invasive ductal carcinoma; grade III with metastatic regional nodes; pTNM - T3 N3 |
| 13 | YBL013 | F/25 | Asian | Ca. Breast | Fibroadenoma,- benign breast lesion - suggestive of fibrocystic change |
| 14 | YBL014 | F/62 | Asian | Ca. Breast | Invasive ductal carcinoma; grade II with reactive regional nodes; pTNM - T1 N0 |
| 15 | YBL015 | F/43 | Asian | Ca. Ovary | Submucousal Polypoid Leiomyoma |
| 16 | YBL016 | F/33 | Asian | Ca. Ovary | Mild Chronic Inflammation |
| 17 | YBL017 | F/55 | Asian | Ca. Breast | Invasive ductal carcinoma; grade III with metastatic regional nodes; pTNM - T3 N3 |
| 18 | YBL018 | F/62 | Asian | Ca. Breast | Invasive ductal carcinoma; grade II with reactive regional nodes; pTNM - T2 N0 |
| 19 | YBL019 | F/72 | Asian | Ca. Ovary | High grade sero-mucinous adenocarcinoma involving both ovaries with metastatic deposit in omentum & reactive regional nodes |
| 20 | YBL020 | F/44 | Asian | Ca. Breast | Invasive ductal carcinoma; grade III with reactive regional nodes; pTNM - T2 N3 |
| 21 | YBL021 | F/55 | Asian | Ca. Breast | Invasive ductal carcinoma; grade III with reactive regional nodes |
| 22 | YBL022 | F/52 | Asian | Ca. Ovary | Moderately differentiated papillary serous adenocarcinoma involving both ovaries infiltrating into colonic wall with metastatic deposit in right & left abdominal pelvic wall peritoneum; pTNM - T4 N2 |
| 23 | YBL023 | F/60 | Asian | Ca. Breast | Invasive ductal carcinoma grade III with reactive regional nodes pTNM-T2 N2 |
| 24 | YBL024 | F/45 | Asian | Ca. Breast | Invasive ductal carcinoma; grade III with metastatic regional nodes; pTNM - T2 N3 |
| 25 | YBL025 | F/51 | Asian | Ca. Ovary | Both ovaries -  corpus albicans and follicular cysts |
| 26 | YBL026 | F/46 | Asian | Ca. Breast | Invasive ductal carcinoma, grade III with reactive regional nodes |
| 27 | YBL027 | F/69 | Asian | Ca. Breast | Invasive ductal carcinoma; grade III with metastatic regional nodes; pTNM - T3 N3 |
| 28 | YBL028 | F/45 | Asian | Ca. Breast | Malignant phyllodes tumor with reactive nodes |
| 29 | YBL029 | F/32 | Asian | Ca. Breast | Benign phyllodes tumor |
| 30 | YBL030 | F/38 | Asian | Ca. Breast | Invasive ductal carcinoma grade III with reactive regional nodes |
| 31 | YBL031 | F/38 | Asian | Ca. breast | Invasive ductal carcinoma grade III with reactive regional nodes |
| 32 | YBL032 | F/71 | Asian | Ca. Breast | Invasive ductal carcinoma |
| 33 | YBL033 | F/18 | Asian | Ca. Ovary | Right ovarian mass - suggestive of gangrenous change |
| 34 | YBL034 | F/63 | Asian | Ca. Breast | Invasive ductal carcinoma; grade III with metastatic regional nodes, pTNM - T2 N3 |
| 35 | YBL035 | F/45 | Asian | Ca. Ovary | Left Ovarian Mass - Mature Teratoma |
| 36 | YBL036 | F/60 | Asian | Ca. Breast | Invasive Ductal Carcinoma Grade III |
| 37 | YBL037 | F/42 | Asian | Ca. Breast | Invasive ductal carcinoma grade III with metastatic unilateral regional nodes pTNM - T2 N1 |
| 38 | YBL038 | F/70 | Asian | Ca. Ovary (Adenoid) | Metastatic deposits in both ovaries with metastatic regional nodes |
| 39 | YBL039 | F/21 | Asian | Ca. Ovary | Metastatic poorly differentiated adenocarcinoma |
| 40 | YBL040 | F/70 | Asian | Ca. Ovary (Adenoid) | Moderately differentiated adenocarcinoma with regional nodes |
| 41 | YBL041 | F/65 | Asian | Ca. Breast | Invasive ductal carcinoma grade II |
| 42 | YBL042 | F/43 | Asian | Ca. Breast | Invasive duct carcinoma, grade III with metastatic regional nodes |
| 43 | YBL043 | F/66 | Asian | Ca. Breast | Invasive ductal carcinoma, grade III with reactive regional nodes |
| 44 | YBL044 | F/65 | Asian | Ca. Breast | Metastatic bilateral regional nodes |
| 45 | YBL045 | F/51 | Asian | Ca. Ovary (Adenoid) | Invasive ductal carcinoma, grade III with reactive regional nodes |
| 46 | YBL046 | F/37 | Asian | Ca. Ovary | Moderately differentiated papillary adenocarcinoma with reactive regional nodes pTNM-pT1N0 |
| 47 | YBL047 | F/58 | Asian | Ca. Breast | Invasive ductal carcinoma; grade III with metastatic regional nodes, pTNM - pT2 N2 |
| 48 | YBL048 | F/75 | Asian | Ca. Ovary (Adenoid) | Invasive ductal carcinoma, grade III with reactive regional nodes |
| 49 | YBL049 | F/74 | Asian | Ca. Breast | Invasive ductal carcinoma, grade III |
| 50 | YBL050 | F/65 | Asian | Ca. Breast | invasive ductal carcinoma, grade III |
| 51 | YBL051 | F/55 | Asian | Ca. Breast | Poorly differentiated adenocarcinoma |
| 52 | YBL052 | F/75 | Asian | Ca. Breast | Invasive ductal carcinoma, grade III with metastatic regional node |
| 53 | YBL053 | F/58 | Asian | Ca. Breast | Invasive ductal carcinoma, grade III with metastatic regional node |
| 54 | YBL054 | F/69 | Asian | Ca. Breast | Invasive ductal carcinoma; grade III with metastatic regional nodes |
| 55 | YBL055 | F/42 | Asian | Ca. Ovary (Adenoid) | Moderately differentiated papillary serous adenocarcinoma involving ovary with metastatic deposits in omentum & reactive regional nodes |
| 56 | YBL056 | F/36 | Asian | Ca. Breast | invasive carcinoma grade II |
| 57 | YBL057 | F/56 | Asian | Ca. Breast | Invasive ductal carcinoma, grade III with metastatic regional node |
| 58 | YBL058 | F/35 | Asian | Ca. Ovary | Moderately differentiated mucinous adenocarcinoma involving right ovary with reactive regional nodes |
| 59 | YBL059 | F/60 | Asian | Ca. Breast | Invasive ductal carcinoma, grade III with metastatic regional node |
| 60 | YBL060 | F/77 | Asian | Ca. Breast | Invasive ductal carcinoma Grade III (T2N3) |
| 61 | YBL061 | F/49 | Asian | Ca. Breast | Invasive ductal carcinoma, grade III with metastatic regional nodes |
| 62 | YBL062 | F/65 | Asian | Ca. Breast | No residual tumor with reactive regional nodes. pTNM - pT2 N2 |
| 63 | YBL063 | F/45 | Asian | Ca. Breast | Grade II with reactive regional nodes. pTNM - pT2 N2 |
| 64 | YBL064 | F/53 | Asian | Ca. Breast | Invasive ductal carcinoma, grade III with metastatic regional nodes |
| 65 | YBL065 | F/58 | Asian | Ca. Ovary | Moderately differentiated mucinous adenocarcinoma involving left ovary with reactive lymph nodes |
| 66 | YBL066 | F/45 | Asian | Ca. Breast | Invasive ductal carcinoma, grade III |
| 67 | YBL067 | F/49 | Asian | Ca. Breast | Mixed mucinous carcinoma, grade III with metastatic regional nodes |
| 68 | YBL068 | F/60 | Asian | Ca. Ovary (Adenoid) | Well differentiated mucinous adenocarcinoma involving one ovary with reactive regional nodes |
| 69 | YBL069 | F/45 | Asian | Ca. Ovary | Both ovaries corpus albicans |
| 70 | YBL070 | F/47 | Asian | Ca. Breast | Invasive ductal carcinoma - grade III with metastatic regional nodes, pTNM - pT2 N1 |
| 71 | YBL071 | F/62 | Asian | Ca. Breast | Invasive duct carcinoma, grade III |
| 72 | YBL072 | F/61 | Asian | Ca. Ovary | Low grade serous adenocarcinoma involving right ovary with reactive regional nodes |
| 73 | YBL073 | F/52 | Asian | Ca. Breast | Invasive ductal carcinoma, grade III,  ER - positive, PR - positive, HER2 - negative |
| 74 | YBL074 | F/23 | Asian | Ca. Ovary (Adenoid) | level 1a - single reactive node |
| 75 | YBL075 | F/59 | Asian | Ca. Ovary | Moderately differentiated serous adenocarcinoma |
| 76 | YBL076 | F/55 | Asian | Ca. Ovary | Invasive lobular carcinoma, grade III with metastatic regional nodes, pTNM - pT2 N2 |
| 77 | YBL077 | F/38 | Asian | Ca. Breast | Invasive ductal carcinoma, grade III with metastatic regional nodes, pTNM - pT2 N2 |
| 78 | YBL078 | F/63 | Asian | Ca. Ovary | Acute inflammation with reactive cellular changes |
| 79 | YBL079 | F/70 | Asian | Ca. Breast | Invasive ductal carcinoma, Grade III |
| 80 | YBL080 | F/74 | Asian | Ca. breast | Invasive tubular carcinoma with reactive unilateral regional nodes |
| 81 | YBL081 | F/68 | Asian | Ca. Breast | Invasive ductal carcinoma, grade III with metastatic regional nodes, pTNM - pT3 N1 |
| 82 | YBL082 | F/47 | Asian | Ca. Breast | Invasive ductal carcinoma, grade III with reactive regional nodes |
| 83 | YBL083 | F/57 | Asian | Ca. Breast | Invasive ductal carcinoma, grade II |
| 84 | YBL084 | F/67 | Asian | Ca. Breast | Metastatic adenocarcinoma without perinodal extension |
| 85 | YBL085 | F/71 | Asian | Ca. Breast | Invasive ductal carcinoma, Grade II with metastatic regional nodes |
| 86 | YBL086 | F/85 | Asian | Ca. Breast | Mucinous carcinoma, Grade II with reactive regional nodes |
| 87 | YBL087 | F/71 | Asian | Ca. Breast | Invasive ductal carcinoma, grade III with reactive regional nodes |
| 88 | YBL088 | F/61 | Asian | Ca. Ovary | spindle cell carcinoma |
| 89 | YBL089 | F/65 | Asian | Ca. Breast | Invasive lobular carcinoma, grade III with reactive regional nodes |
| 90 | YBL090 | F/47 | Asian | Ca. Ovary | Squamous metaplasia & mild chronic cervicitis |
| 91 | YBL091 | F/47 | Asian | Ca. Ovary | Moderately differentiated papillary serous adenocarcinoma involving ovary with reactive regional nodes |
| 92 | YBL092 | F/73 | Asian | Ca. Breast | Ductal carcinoma in situ (DCIS) |
| 93 | YBL093 | F/63 | Asian | Ca. Breast | Invasive ductal carcinoma, grade III with metastatic regional nodes |
| 94 | YBL094 | F/72 | Asian | Ca. Breast | Invasive ductal carcinoma, grade II |
| 95 | YBL095 | F/79 | Asian | Ca. Ovary (Adenoid) | Right ovarian mass granulosa cell tumer pTNM-pT1N0 |
| 96 | YBL096 | F/38 | Asian | Ca. Breast | Invasive ductal carcinoma grade III with reactive regional nodes pTNM-pT4N0 |
| 97 | YBL097 | F/51 | Asian | Ca. Ovary Mass | Well differentiated mucinous adenocarcinoma |
| 98 | YBL098 | F/46 | Asian | Ca. Ovary (Adenoid) | Well two moderately differentiated papillary serous adenocarcinoma involving both ovaries with metastatic deposits |
| 99 | YBL099 | F/61 | Asian | Ca. Breast | Metaplastic carcinoma grade III with metastatic regional node pTNM - pT4 N1 |
| 100 | YBL100 | F/54 | Asian | Ca. Breast | Invasive duct carcinoma grade III with metastatic region node pTNM - pT4 N2 |
| 101 | YBL101 | F/75 | Asian | Ca. Breast | Invasive duct carcinoma grade III |
| 102 | YBL102 | F/34 | Asian | Ca. Ovary | Granulosa cell tumour pTNM - pT4 N2 |
| 103 | YBL103 | F/55 | Asian | Ca. Breast | Invasive duct carcinoma grade III |
| 104 | YBL104 | F/57 | Asian | Ca. Ovary | Moderately differentiated papillary serous adeno carcinoma involving ovary with reactive regional nodes. |
| 105 | YBL105 | F/48 | Asian | Ca. Breast | Invasive duct carcinoma grade III with reactive regional nodes pTNM-pT3N0. |
| 106 | YBL106 | F/44 | Asian | Ca. Ovary | Moderately differentiated papillary serous adeno carcinoma involving ovary with reactive regional nodes. |
| 107 | YBL107 | F/56 | Asian | Ca. Breast | Metaplastic carcinoma grade III with reactive regional nodes.pTNM-pT3N0 |
| 108 | YBL108 | F/55 | Asian | Ca. Breast | Invasive duct carcinoma grade III with reactive regional nodes.pTNM-pT3N0 |
| 109 | YBL109 | F/52 | Asian | Ca. Ovary (Adenoid) | Low grade papillary serous adeno carcinoma involving both ovary with reactive regional nodes. pTNM-pT1C2pN0 |
| 110 | YBL110 | F/56 | Asian | Ca. Breast | Invasive breast carcinoma grade III with metastatic regional nodes, pTNM-pT2N3 |
| 111 | YBL111 | F/47 | Asian | Ca. Breast | Invasive breast carcinoma grade III with metastatic regional nodes pTNM- pT2N3 |
| 112 | YBL112 | F/41 | Asian | Ca. Breast | Invasive duct carcinoma grade III with metastatic regional nodes pTNM-PT4N3 |
| 113 | YBL113 | F/72 | Asian | Ca. Ovary (Adenoid) | Low grade adenocarcinoma involving left ovary |
| 114 | YBL114 | F/46 | Asian | Ca. Ovary | Moderately differentiated adenocarcinoma involving both ovaries |
| 115 | YBL115 | F/69 | Asian | Ca. Ovary (Adenoid) | Left ovarian mass: high grade adenocarcinoma involving left ovary. |
| 116 | YBL116 | F/74 | Asian | Ca. Breast | Invasive breast carcinoma grade III with metastatic regional nodes. pTNM- pT2N1 |
| 117 | YBL117 | F/59 | Asian | Ca. Ovary | Moderately differentiated papillary serous adenocarcinoma involving ovary with reactive regional nodes |
| 118 | YBL118 | F/56 | Asian | Ca. Breast | Invasive breast carcinoma grade III with metastatic regional nodes pTNM-pT2N1 |
| 119 | YBL119 | F/55 | Asian | Ca. Ovary | Moderately differentiated adenocarcinoma involving both ovaries |
| 120 | YBL120 | F/56 | Asian | Ca. Breast | Invasive breast carcinoma grade III with metastatic regional nodes pTNM-pT3N1 |
| 121 | YBL121 | F/69 | Asian | Ca. Breast | Invasive ductal carcinoma grade III with reactive regional nodes pTNM-pT3N0 |
